# Effect of Surface Finishing State on Fatigue Strength of Cast Aluminium and Steel Alloys

**DOI:** 10.3390/ma16134755

**Published:** 2023-06-30

**Authors:** Matthias Oberreiter, Michael Horvath, Michael Stoschka, Stefan Fladischer

**Affiliations:** 1 Christian Doppler Laboratory for Manufacturing Process Based Component Design, Chair of Mechanical Engineering, Montanuniversitaet Leoben, Franz-Josef-Strasse 18, 8700 Leoben, Austria; michael.horvath@unileoben.ac.at (M.H.); michael.stoschka@unileoben.ac.at (M.S.); 2 Chair of Mechanical Engineering, Montanuniversitaet Leoben, Franz-Josef-Strasse 18, 8700 Leoben, Austria; stefan.fladischer@unileoben.ac.at

**Keywords:** fatigue assessment, residual stress, bulk defects, surface defects, computed tomography, elastic–plastic behaviour, fracture mechanics, cast steel, cast aluminium, vibratory finishing

## Abstract

The endurance limit of structural mechanical components is affected by the residual stress state, which depends strongly on the manufacturing process. In general, compressive residual stresses tend to result in an increased fatigue strength. Post-manufacturing processes such as shot peening or vibratory finishing may achieve such a compressive residual stress state. But within complex components, manufacturing-process-based imperfections severely limit the fatigue strength. Thus, the interactions of imperfections, residual stress state and material strength are key aspects in fatigue design. In this work, cast steel and aluminium alloys are investigated, each of them in vibratory finished and polished surface condition. A layer-based fatigue assessment concept is extended towards stable effective mean stress state considering the elastic–plastic material behaviour. Murakami’s concept was applied to incorporate the effect of hardness change and residual stress state. Residual stress relaxation is determined by elastic–plastic simulations invoking a combined hardening model. If the effective stress ratio within the local layer-based fatigue strength is evaluated as critical distance value, a sound calculation of fatigue strength can be achieved. Summing up, the layer-based fatigue strength design is extended and features an enhanced understanding of the effective stabilized mean stress state during cyclic loading.

## 1. Introduction

Due to the continuously increasing requirements of our modern industrial market, focus is laid on economic and sustainable design. Lightweight engineering is often driven by topology optimization, yielding to complexly shaped components [1]. Due to benefits in castability and comparable low density, AlSi-cast alloys are broadly used in mobility industry and transport applications [2,3,4]. Besides Al-alloys, cast steel alloys play an important role as well due to improved structural load-bearing capability [5,6,7]. The geometrical complexity of cast parts results in high local load factors. Moreover, strongly varying microstructural conditions may occur due to changes in local process parameters like local solidification [8,9,10]. Such local microstructural conditions lead to varying shape and degree of porosity, affecting the local acting stress field significantly [11,12,13]. This results in a severely limited fatigue strength of imperfective cast components compared to the strength of the ideal, defect free material [2,14,15,16,17,18,19]. From the point of manufacturing quality, porosity can be subdivided into gas and shrinkage porosity according to [20] and may be determined either destructively by fractographical inspection or non-destructively via computed tomography (CT) [21,22,23].

Besides inhomogeneities, the effective mean stress state plays an important role in cyclic loading [24,25,26,27]. Although the global cyclic load predominantly results in elastic deformation, the aforementioned defects may lead to localized cyclic plasticisation and provoke therefore a change of the effective mean stress state [28,29]. In order to improve fatigue life of components, one can either reduce the external load, or improve the resistance of the material. If the material cannot be changed, a modification of the local strength by mechanical, chemical or thermal treatment may be helpful. For example, hardening or deep rolling of steel alloys leads to improvement of hardness and to compressive residual stresses depending on the process settings [30,31]. Shot peening yields an increased compressive residual stress state and hardness as well, but increases surface roughness [26,32,33,34,35]. Moreover, welded structures can be treated by HFMI to improve fatigue strength due to local work hardening and compressive residual stresses [36,37]. In contrast, surface treatment by electrochemical polishing leads to a high surface quality but does not evoke residual stresses [38]. Recent additive manufacturing technologies (3D/4D printing) feature multi-material composite design enabling tailored layer-based properties [39]. Residual stresses can be induced by other post treatment processes as well [40,41,42,43,44,45,46]. In detail, grinding and polishing by vibratory finishing as post-manufacturing process can be performed on simple fatigue specimens, but is also well applicable to complex parts like turbines blades [47,48,49]. Vibratory finishing as a post manufacturing process leads to high reproducibility as well as a good surface quality with low scatter of surface roughness values as function of the process settings [50,51,52]. But the fatigue design may be affected by this highly reproductive finishing processes due to the evocation of compressive residual stresses in the surface layer [47,53,54] and a change of surface layer hardness due to small plastic deformations [54,55], depending on the material as well as the polishing medium. These effects of vibratory processing lead often to an increase of the long life fatigue strength [56], but may also reduce fatigue strength [57] depending on the effective residual stress state in the surface layer. Such contrary statements regarding the effects of post-manufacturing surface treatments highlight the importance of considering the effective mean stress state within the most-stressed layer properly.

Regarding nominal fatigue assessment of imperfective material, the common engineering guideline FKM (Forschungskuratorium Maschinenbau) recommends safety factors depending on the methods of non-destructive testing during quality control, but does not consider the statistical distribution of defects within the components [58]. Surface treatment processes are incorporated as surface-layer-factor for post-treatments like hardening, deep rolling or shot peening for example, but the effective residual stress state is not taken into account in detail within this guideline. Application of such general safety factors leads to an underestimation of the fatigue strength, and therefore to a contradiction to modern light-weight design goals.

To assess the fatigue strength of notches or defects individually, the Theory of Critical Distances (TCD) by Taylor offers a widely used local methodology [59,60]. Within this approach, three methods are formulated, namely point, line and area methods. The point method states that the linear-elastic stress at a certain distance (critical distance) from the hot-spot is equal the fatigue strength of plain specimens $\Delta \sigma_{0}$. El-Haddad et al. [61] introduced the material intrinsic length $a_{0}$ as a function of $\Delta \sigma_{0}$ and the long crack threshold $\Delta K_{th,lc}$; see Equation (1). Taylor’s critical distance is proportional to the intrinsic length of El-Haddad, as stated in [59]. The proposed methodology was applied and validated in several studies [62,63,64] with regard to notched components. Moreover, Taylor’s introduced methods were successfully applied considering the residual stress state in the surface layer of shot peened specimens [32,33]. Therein, sound predictions of the fatigue strength could be observed considering the residual stress state at a critical distance (point method) [65], or by averaging the stress distribution over a distance from the surface (line method) [32,33,34,35].

(1) $$a_{0}=\frac{1}{\pi }\cdot {\left(\frac{\Delta K_{th,lc}}{\Delta \sigma_{0}}\right)}^{2}$$

In order to determine the cyclic stable stress state of ductile materials containing defects and residual stresses, a detailed knowledge about the elastic–plastic material behaviour is necessary. Due to the non-linear material response in parts loaded above a certain stress level, work hardening and subsequent mean stress relaxation occurs [66,67]. This implies that the cyclic loading hysteresis is shifting until stabilization of the material behaviour is reached [68,69]. As the distributed defects may vary severely in shape and size within the highly-stressed volume, the effective load stress due to this stabilization procedure of the hysteresis loop, the effective load–stress ratio $R_{eff}$ is introduced as a function of residual stress profile and external loading [66,70,71]. The stabilization process of the residual stress profile in the surface layer has been well studied for welds [72,73,74], shot peened parts [75,76,77] or in the case of additive manufactured structures [24].

In order to consider the non-linear cyclic deformation behaviour, a mathematical connection between stresses and strains is provided by the constitutive material equation [78]. One common model to describe the elastic–plastic behaviour is provided by Chaboche [79]. This combined hardening model includes an isotropic and kinematic part, and is based on a yield function *f*, given in Equation (2). Within this equation, the von Mises criterion is denoted by the term J(σ−χ), specified in Equation (3) [79].

(2) f=J(σ−χ)−R−k

(3) $$J\left(\sigma -\chi \right)=\sqrt{\frac{3}{2}\cdot \left(\sigma^{\prime }-\chi^{\prime }\right):\left(\sigma^{\prime }-\chi^{\prime }\right)}$$

In Equation (3), the variable σ describes the stress tensor and χ is the kinematic back stress tensor; $\sigma^{\prime }$ and $\chi^{\prime }$ are the related deviatoric components. The variable *k* represents the initial size of the yield surface and the isotropic part *I* of the combined hardening model describes the evolution of the size of the surface, as given in Equation (4). Plastic deformation of the component takes place if the yield function reaches the limit value of zero, while f<0 indicates a linear elastic material behaviour [79].

(4) $$I=Q\cdot \left(1-e^{-b_{s}\cdot p}\right)$$

The change of the size of the yield surface is described by isotropic hardening (see Equation (4)) as a function of the accumulated plastic strain, but not referring to the position in the stress space. The constants *Q* and $b_{s}$ describe the difference between the initial and stabilized size of the asymptotic surface value and the stabilization rate as function of the accumulated plastic strain *p* [78,80].

(5) $$\chi =\sum_{i=1}^{m}\chi_{i}$$

The translation of the yield surface in the stress space is given by the kinematic hardening model keeping the size of the yield surface constant, represented by the kinematic stress tensor χ in Equation (5) [79]. For uniaxial loading, a non-linear approach can be expressed (see Equation (6)) with the constants $C_{i}$ and $\gamma_{i}$ [79]. The accuracy of the model can be improved by superimposing higher order approaches $\chi_{i}$, as indicated by the variable m according to Equation (5).

(6) $$\chi_{i}=\psi \frac{C_{i}}{\gamma_{i}}+\left(\chi_{0,i}-\psi \frac{C_{i}}{\gamma_{i}}\right)e^{-\psi \gamma_{i}\left(\epsilon_{p}-\epsilon_{p,0}\right)}$$

The initial conditions are represented by the parameters $\chi_{0,i}$ and $\epsilon_{p,0}$ in Equation (6) and the value ψ describes the flow direction of the yield surface [79]. $C_{i}$ and $\gamma_{i}$ representing the kinematic part as well as *Q* and *b* for the isotropic part can be experimentally determined based on low-cycle fatigue (LCF) tests using an optimisation routine, proposed in [81].

For ductile metal structures of engineering components containing manufacturing-process-based inhomogeneities, fracture mechanics approaches feature reliable design tools to estimate the fatigue strength [3,82,83,84,85,86,87,88]. According to Murakami [21], defects can be treated as cracks with the size $\sqrt{\mathrm{area}}$ of the projected convex hull independent from their morphology. The influence of the defect’s location is introduced by means of a geometry factor *Y* [89]. Kitagawa and Takahashi [90] introduced a threshold based methodology to describe the endurance fatigue strength as a function of the defect size, Equation (7). Related extensions of the Kitagawa–Takahashi diagram (KTD) according to El-Haddad introduce a smooth transition between long and short crack behaviour, and Chappetti’s extension incorporates the formation of crack closure effects respectively [61,91]. Maierhofer et al. [92] recommends describing the crack extension from the intrinsic short crack threshold $\Delta K_{th,eff}$ up to the long crack threshold $\Delta K_{th,lc}$ by Equation (8) as functions of the predominant closure mechanisms *i*. Regarding the in-depth influence of microstructure on crack growth parameters and the effect of the highly stressed volume, the authors refer to previous investigations [93,94] for Al–Si cast alloys.

(7) $$\Delta \sigma_{LLF}=\frac{\Delta K_{th,\Delta_{a}}}{Y\cdot \sqrt{\pi \cdot a}}$$

(8) $$\Delta K_{th,\Delta a}=\Delta K_{th,eff}+\left(\Delta K_{th,lc}-\Delta K_{th,eff}\right)\left[1-\sum_{i=1}^{n}\nu_{i}\cdot \exp\left(-\frac{\Delta a}{l_{i}}\right)\right]$$

with

$$\sum_{i=1}^{n}\nu_{i}\equiv 1$$

Moreover, the threshold based tool of Kitagawa and Takahashi was applied in [24,95] for superimposing load and residual stresses and is quite effective for fatigue strength assessment considering residual stress measurements. Several studies conclude that residual stresses play a key role in affecting fatigue strength, and respectively crack propagation, in the high cycle fatigue (HCF) region [36,96,97,98,99]. By superimposing the cyclically applied load and residual stress components, an effective load–stress ratio arises, which can strongly differ from the nominal load–stress ratio. This effective local load–stress ratio directly affects the local crack growth behaviour as the effective residual stress state opens or closes the crack flanks [100,101,102]. To consider the influence of the load–stress ratio on the long crack threshold values, Newman [103] introduced an engineering-feasible approach based on an initial crack threshold value $\Delta K_{th,lc,R=0}$ at tumescent load and a constant $C_{th}$, applied for steel in [92] and for aluminium alloys in [93]. In case of a defect free material, representing the upper border of the KTD, the effect of the load–stress ratio can be considered using the nominal stress approach of the common engineering guideline [58]. The fatigue strength of the defect-free material $\Delta \sigma_{LLF,0}$ can be obtained experimentally using hot isostatic pressed (HIP) material, or otherwise estimated based on low cycle fatigue (LCF) tests [104,105]; see Equation (9).

(9) $$\Delta \sigma_{LLF,0}=2\cdot K^{\prime }\cdot {\left(0.0005\right)}^{n^{\prime }}$$

The size of the fracture-initiating defects, representing the axis of abscissae in the KTD, can be described by extreme value statistics, such as the generalized extreme value distribution (GEV) (see Equation (10)) or the derived distributions according to Tiryakioğlu [106]. Extreme value statistics are proved well suited for assessment of extremal defects in components, as summarized by Beretta [107]. But one remaining challenge regarding assessment of extremal defects using computed tomography is the selection of a proper threshold value, which was investigated by Romano et al. [108] using mean excess plots for peak over threshold (POT) sampling.

(10) $$P\left(\sqrt{area},\mu ,\delta ,\xi \right)=\exp\left\{-{\left[1+\xi \left(\frac{\sqrt{area}-\mu }{\delta }\right)\right]}^{-\frac{1}{\xi }}\right\}$$

Besides the Kitagawa–Takahashi diagram, Murakami [21] introduced the well-known $\sqrt{\mathrm{area}}$-concept as an engineering-feasible methodology for fatigue strength calculation of defect-afflicted components using the projected area of inhomogeneities. The original approach developed for steel alloys was extended by Noguchi et al. [109] for aluminium alloys using the ratio of the Young’s modulus, as depicted in Equation (11). The load–stress ratio is considered within the $\sqrt{\mathrm{area}}$-concept as a function of the value α, which depends on the hardness of the material. In case of defect free material, the base material strength can be calculated to 1.6·HV [21].

(11) $$\sigma_{LLF}\left(\sqrt{area},R\right)\;=\;C_{1}\cdot \left(\frac{HV+C_{2}\cdot \frac{E_{Al}}{E_{St}}}{{\left(\sqrt{area}\right)}^{\left(1/6\right)}}\right)\cdot {\left[\frac{1-R}{2}\right]}^{\alpha }$$

with

$$\alpha =0.226+HV\cdot 10^{-4}$$

This paper deals with the influence of vibratory finishing process and associated residual stress state on corresponding layer fatigue strength of three investigated cast materials, which covering steel and aluminium alloys with different manufacturing conditions. Five specimen series were taken from these materials, each of them in polished and vibratory finished condition to enable a direct comparison of the fatigue properties. After a detailed description of the experimental framework, the layer-based fatigue assessment concept, originally published by the authors in [94] is extended with regard to the effective residual stress state within the surface layer evoked by vibratory finishing. The residual stress state in the surface layer is measured using X-ray diffraction. In order to determine the effective stable residual stress state after several load cycles, elastic–plastic simulations are set-up to analyse the material behaviour in the surface layer using a combined hardening model for each material. These results extend the probabilistic fatigue assessment methodology for aluminium alloy EN AC 46200 in T6 and HIP+T6 conditions. Subsequently, the extended methodology featuring a layer-based fatigue design with effective stress ratio dependency is validated for two cast steel alloys. Additionally, Murakami’s approach is applied to study the effect of increased surface micro-hardness by vibratory polishing. Summing up, this paper provides a detailed fatigue strength design method evaluating the effective stress ratio within the highly-stressed layers and thereby demonstrating the benefit of vibratory finishing as additional post-treatment for these cast alloys. If the fracture mechanical and fatigue strength parameters as well as imperfection distributions are known, the layer-based fatigue design method featuring the effective stress ratio $R_{eff}$ can be well applied to other materials and load-cases as well.

## 2. Materials and Methods

**Figure 1:**
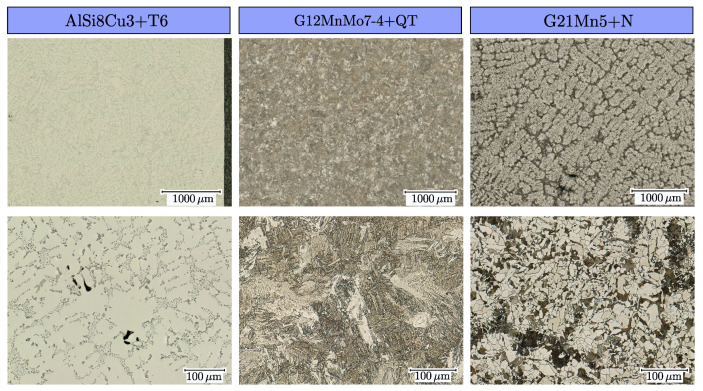
Overview of microstructure for the investigated materials AlSi8Cu3+T6, G12MnMo7-4+QT and G21Mn5+N.

The investigated materials in this study are the cast aluminium alloy AlSi8Cu3 (EN AC 46200) in T6 and HIP+T6 heat treatment conditions and the cast steel alloys G12MnMo7-4+QT in quenched and tempered condition, and G21Mn5+N in normalized condition. The material AlSi8Cu3 was taken out of a gravity cast component which was manufactured by a core package casting process [110]; nominal chemical composition is given in Table 1 following the standard [111]. After the casting process, the components were T6 heat treated to obtain a maximum in ultimate strength. Furthermore, some of the components were HIP+T6 treated to obtain a defect-free material batch. Two different positions were investigated within the components, namely position AlSi8Cu3-A possessing a secondary dendrite arm spacing of $\lambda_{2}=24$ µm in T6 and HIP+T6 condition and position AlSi8Cu3-B with $\lambda_{2}=55$ µm in T6 condition. The secondary dendrite arm spacing is not affected by the HIP processing, as the final T6 condition is identical for both manufacturing routes. Tensile tests revealed a yield strength of YS=153 MPa and tensile strength of TS=326 MPa for position AlSi8Cu3-A, and a yield strength of YS=129 MPa and tensile strength of TS=216 MPa for position AlSi8Cu3-B. It is important to note that the yield strength of YS was evaluated at a strain value of 0.01% due to the brittle behaviour of position AlSi8Cu3-B. An overview of the dendritic microstructure in position AlSi8Cu3-A is given in the left column of Figure 1 in the polished state of the metallographic sample.

A dedicated casting geometry was used for the material G21Mn5+N and G12MnMo7-4+QT to ensure a low degree of shrinkage porosity similar to the standard [112]. The chemical composition of the material G21Mn5+N in normalized condition following the standard SEW 685 [113] is given in Table 2, as well as for material G12MnMo7-4+QT according to the standard SEW 520 [114].

Quasi-static tensile tests for the material G21Mn5 revealed a upper yield strength of UYS=376 MPa, a lower yield strength of LYS=323 MPa, an ultimate tensile strength of UTS=524 MPa and an elongation at fracture of more than 50%. An overview of the dendritic, ferrite–pearlite microstructure is given in Figure 1 in the right column. The results of the quasistatic tensile tests of the high-strength cast steel alloy G12MnMo7-4+QT are given in a preceding work of the authors [115]; the corresponding microstructure as a result of the heat treatment is given in the middle column in Figure 1. The metallographic specimens of the cast steel alloys were grinded and polished, followed by etching with with 3% Nital. After the casting process, all cast parts were pre cut using water jet cutting and finally turned by a CNC machine. The final post-processing of the specimen surfaces, e.g., either manually polished or vibratory finished, is applied as final manufacturing step for all fatigue test samples.

### 2.1. Vibratory Finishing

It is well known that fatigue specimens have to be polished after the CNC-turning process to comply with the specifications of the standard [116,117]. This process is typically carried out manually, but in order to reduce the human influence for highly reproducible surface properties, vibratory finishing appears to be a useful alternative. In addition, electrochemical polishing is an alternative to manual polishing, but requires additional etching equipment and high-current power sources [38]. Whereas polishing primarily targets a reduction of the surface roughness, vibratory finishing offers as side effect a modification of the surface layer regarding residual stress state and hardness distribution, as introduced in Section 1.

**Figure 2:**
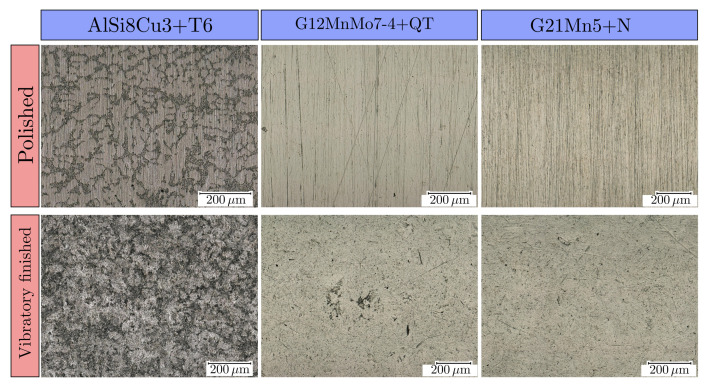
Comparison of vibratory finished and polished surface for the investigated materials AlSi8Cu3+T6, G12MnMo7-4+QT and G21Mn5+N.

A trough vibrator AVAtec^®^ TV 60 (Avatec Gmbh, Sindelfingen, Germany) was used to obtain a defined residual stress state by vibratory finishing. Therefore, different abrasive ceramic and plastic media were used in three subsequent treatment steps (strong grinding, smoothing and polishing). Additionally, a corrosion-preventing compound is necessary to absorb the abrasion of grinding media and workpieces. The specimens were grinded for several hours in the oscillating bowl for each abrasive media. Figure 2 illustrates the obtained surface quality for the polished and vibratory finished condition of each series. Roughness parameters were evaluated with a Mahr^®^ MarSurf VD140 (Mahr Austria GmbH, Vienna, Austria). The arithmetic mean roughness value is $R_{a}=0.095$ µm for the polished (POL) specimens and $R_{a}=0.163$ µm for the vibratory finished (VF) ones, and the total height of the roughness profile is $R_{z}=0.61$ µm for polished and $R_{z}=1.11$ µm for vibratory finished specimens. Due to this quite high surface quality and the low difference of the roughness values, the effect of roughness on fatigue strength was purposely not taken into account in this study. For further sample designation, polished specimens are labelled with the suffix POL and vibratory finished ones with the suffix VF.

### 2.2. High Cycle Fatigue Tests (HCF)

**Figure 3:**
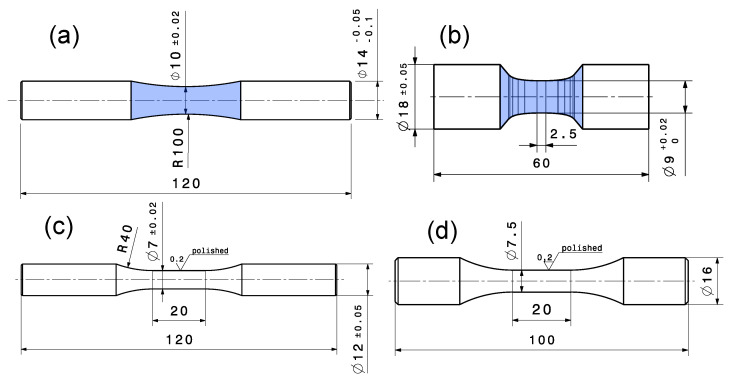
Specimen geometries for fatigue testing: (**a**) HCF steel alloys; (**b**) HCF aluminium alloy; (**c**) LCF steel alloys; (**d**) LCF aluminium alloy. All dimensions are in mm.

The long life fatigue strength $\sigma_{LLF}$ for the investigated specimen series has been determined by uniaxial fatigue testing at room temperature following the standard [118,119]. The force-controlled high-cycle fatigue tests are conducted on a RUMUL^®^ Testonic 150 kN (Russenberger Prüfmaschinen AG, Neuhausen am Rheinfall, Switzerland) resonance test rig for the steel specimens and a RUMUL^®^ Microtron 20 kN (Russenberger Prüfmaschinen AG, Neuhausen am Rheinfall, Switzerland) for the aluminium specimens, imposing a global nominal load ratio of R=−1. Figure 3a,b depict the specimen geometries used for high cycle fatigue (HCF) testing, which are manufactured from bulk material. The blue marked test sections in Figure 3 were finished with the respective surface treatment, either manually polished or vibratory finished. Each test terminated with the final rupture of the specimen, respectively a certain drop in frequency, or if the run-out number of $1\times 10^{7}$ cycles is achieved. In absence of any damage, in terms of observable surface crack initiation, the run-outs were subsequently retested at high stress levels in the finite life region of the S/N-curve to study the fracture initiating defects for all fatigue samples. Table 3 gives an overview of the high cycle fatigue tests with the related manufacturing and post-treatment coding. It should be mentioned that a part of these summarized results was obtained in preceding work by the authors [115,120,121].

### 2.3. Low Cycle Fatigue Tests (LCF)

Low cycle fatigue experiments were conducted for the investigated cast materials to determine the elastic–plastic behaviour. The specimens were manufactured following the standard [116,122] featuring only a polished surface condition; see Figure 3c for steel alloys and Figure 3d for aluminium alloy. A servo-hydraulic test rig was used for the strain-controlled fatigue tests utilizing an extensometer for strain measurement. The specimens are tested at a triangular loading sequence featuring a constant strain rate of 1% per second at an alternate strain ratio of $R_{\epsilon }=-1$.

### 2.4. Residual Stress Measurements

Polished and vibratory finished specimens of both steel series were subjected to residual stress measurements conducted with a X-RAYBOT from MRX-RAYS^®^ (MRX-RAYS, Brumath, France). This portable X-ray diffractometer was equipped with a 2 mm collimator in accordance with the standard DIN EN 15305 [123], limiting the radius of the irradiated area to the half radius of the surface curvature. The aluminium series were measured with a Seifert XRD Charon SXL (XRD Eigenmann GmbH, Schnaittach, Germany) by Materials Center Leoben Forschung GmbH with a 0.3 mm collimator. Cr-Kα radiation was used for all series with a wavelength of λ=2.291 Å and a ψ-mounting measurement setup following the standard [124]. Furthermore, the applied measurement procedure is in accordance with the standard ASTM E2860-12 [125]. A V-foil is placed in the secondary optical path to filter the Cr-radiation. The X-ray tube is operating at 20 kV and 1 mA and tilts in a range of ±40° in axial specimen direction (ψ-angle). A total number of 17 increments with an exposure time of 60 s each point is used. For the residual stress determination, the diffraction peaks from Al-alpha 311 and Fe-alpha 211 were used and the detected peak shape is fitted by means of a Pseudo-Voight function. The stress evaluation is performed based on the $2\theta -sin^{2}\left(\psi \right)$ method and elliptical fitting of the data points at a confidence level of 90% is applied. All residual stresses (surface and gradient in depth) were measured along the specimen axis, which represents the loading direction, at selected samples of each material batch.

**Figure 4:**
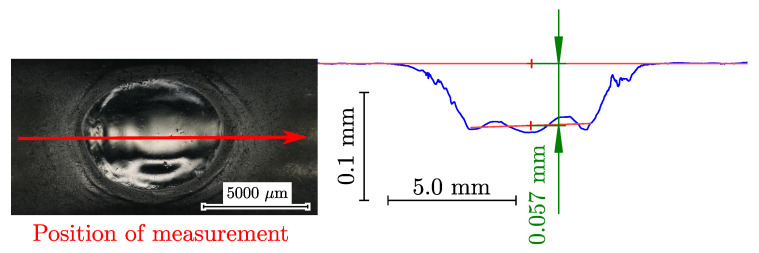
Electrolytic polished surface on a steel specimen (**left**) and corresponding cross-section (**right**). The red arrow represents the direction of measurement for the blue profile.

To carefully remove small layers from the surface a Struers^®^ LectroPol-5 (Struers GmbH, Willich, Germany) was used for electrolytic polishing, exemplarily depicted as polished notch at the specimen surface in Figure 4. A 3D-printed, flow-optimized fluid directing mask characterised by a 0.5 ${\mathrm{cm}}^{2}$ circular area was used at a voltage of 36 V for polishing in the test-cross section of the specimen. The electrolytic solution A2 of Struers is used at a temperature of 5 °C up to 10 °C to perform a constant material removal rate. The electrochemical erosion time was set for each increment individually, guaranteeing that the target depth was reached for subsequent residual stress measurements. The depth profile was measured by a Mahr^®^ MarSurf VD140 (Mahr Austria GmbH, Vienna, Austria) using the contour measuring unit, yielding a depth profile as depicted in Figure 4.

### 2.5. Fracture Analysis and Computed Tomography

Subsequently to the fatigue testing, the ruptured specimens’ fracture surfaces were analysed utilizing a digital optical microscope (DOM) from KEYENCE^®^ VHX-5000 (Keyence Corporation, Osaka, Japan) for macroscopic inspection. Local analysis of the fracture initiating defect was carried out by means of a ZEISS EVO 15 (Carl Zeiss AG, Oberkochen, Germany) scanning electron microscope (SEM), allowing a further magnified inspection of fracture origin and measurement of defects’ sizes. Non-destructive testing by means of computed tomography (CT) was realized by a Phoenix/X-ray Nanotom 180 (GE inspection technologies, Lewistown, PA, USA) using a voxel-size of just 4.5 μm for series AlSi8Cu3-A-T6 and 5.5 μm for AlSi8Cu3-B-T6.

### 2.6. Hardness Measurement

Macro hardness was measured by a ZWICK^®^ ZHU 2.5 (ZwickRoell GmbH & Co. KG, Ulm, Germany) test rig using a Vickers indenter. The applied testing parameters correspond to HV1 testing, according to standard ASTM E384 [126], and were used for surface hardness measurements to differentiate between polished and vibratory finished condition. Furthermore, nano-indentation measurements were conducted at the Department of Materials Science (Montanuniversität Leoben, Leoben, Austria) for selected specimens to determine a work hardening of the surface layer by post treatment. Therefore, a Keysight Nanoindenter G200 (Keysight Technologies, Santa Rosa, CA, USA) testing unit utilizing a Berkovich indenter at a constant strain rate of 0.05 $s^{-1}$ was used. The indentation depth was 150 nm for cross section in the surface layer and 2500 nm for the cross section in the bulk material.

### 2.7. Crack Propagation Testing

**Figure 5:**
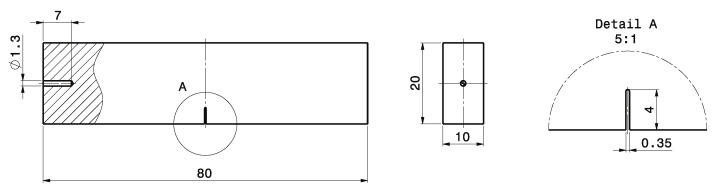
SENB-specimen geometry for fatigue crack growth testing. All dimensions are in mm.

To build up the Kitagawa–Takahashi diagram and its fracture mechanical extensions, detailed information about the crack growth behaviour is necessary. Long-crack growth threshold stress intensity factors $\Delta K_{th,lc}$ were determined for load ratios from R=−1 up to R=0.8 to obtain the Newman curve [103]. The short crack threshold values $\Delta K_{th,eff}$ were determined at high load–stress ratios to minimize closure effects, as presented in in [127,128]. Single edge notched bending specimens were utilized. Their geometry is illustrated in Figure 5.

After extracting the specimens from the bulk material using a CNC-milling machine, an initial notch featuring a depth of 4 mm and a width of 0.35 mm was manufactured using wire cut discharge machining (EDM). To facilitate the measurement of the initial notch geometry by the DOM, the sides of the sample were grinded and polished up to 1 μm using diamond suspension. A sharp mechanical notch, which is necessary for subsequent compression pre-cracking, was produced by razor-blade polishing utilizing a 6 μm diamond paste. Compressive pre-cracking [129,130] was conducted on a SincoTec^®^ Power Swing MOT100kN (SincoTec Test Systems GmbH, Clausthal-Zellerfeld, Germany) resonance test rig at a stress ratio of R=20. To determine the cyclic R-curve behaviour, the stepwise rising load amplitude crack growth test according to Tabernig et al. [131] was applied. Crack propagation experiments were conducted using a RUMUL^®^ Cracktronic (Russenberger Prüfmaschinen AG, Neuhausen am Rheinfall, Switzerland) resonance test rig. The constant amplitude loading was started at $\Delta K<\Delta K_{th,eff}$ and stepwise increased to capture the cyclic R-curve until long-crack propagation $\Delta K>\Delta K_{th,lc}$ occurred. The crack length was measured by the direct current potential voltage drop (DCPD) method incorporating a temperature coefficient to compensate the effect of ambient temperature change.

## 3. Results

### 3.1. High Cycle Fatigue Strength

The statistical evaluation of the fatigue data in the finite life region was conducted according to the standard [132]. In the long-life fatigue regime, the fatigue strength was estimated at a probability of survival of $P_{S}=50\%$ applying the $arcsin(\sqrt{P})$ transformation [133]. The slope $k_{2}$ in the long-life region was estimated as function of the slope in the finite life region $k_{2}=5\cdot k_{1}$, as proven applicable in [19,134]. Some of the fatigue test results can be found in previous publications [120,135], but additional data points were included to extend the experimental database and improve statistical evaluation.

**Figure 6:**
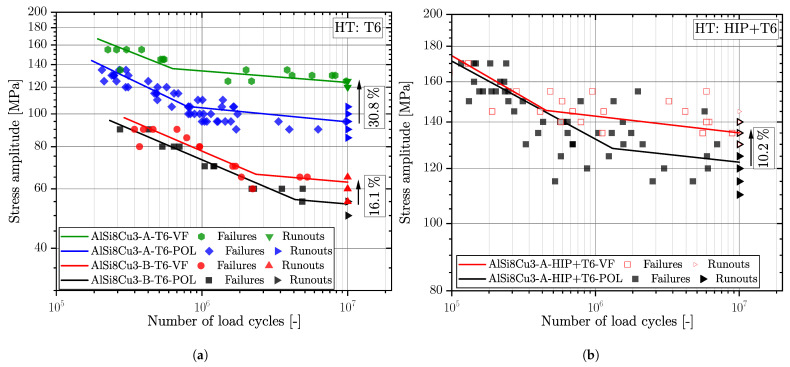
S-N-curves for (**a**) T6 heat treated and (**b**) HIP+T6 heat treated cast material AlSi8Cu3 in vibratory finished and polished surface condition for load–stress ratio R=−1.

Figure 6 displays the fatigue data of the material AlSi8Cu3 in heat treatment T6 (a) and HIP+T6 (b) condition. Each test series of the Al-specimens is opposed for the two different surface conditions; see suffix POL and VF. For the HIP+T6 treated series, representing the porosity free base material strength, the long life fatigue strength of the vibratory finished samples (AlSi8Cu3-A-HIP+T6-VF) was increased by 10.2% compared to the polished ones (AlSi8Cu3-A-HIP+T6-POL); see S-N curves in Figure 6b. The slopes in the finite life region for both surface states are quit similar, but the scatter index of the vibratory finished samples is significantly reduced compared to the polished state; see summary in Table 4.

Series AlSi8Cu3-A-T6 revealed a improvement of the mean long life fatigue strength due to vibratory finishing of 30.8%; see blue and green marked data in Figure 6a. This position revealed a decrease in the scatter index in the long life region too, as well as the slope in the finite life region keeps similar. Concerning the fatigue data for the coarse microstructure, represented by series AlSi8Cu3-B-T6, the slope $k_{1}$ for polished is along the slope for vibratory finished surface, but the scatter index is increased for the vibratory finished fatigue data. Apart the high porosity of these samples the comparable low number of tested fatigue specimens might be a reason for the slightly increased fatigue scatter index. An increase in mean fatigue strength was additionally achieved by 16.1% by vibratory finishing.

**Figure 7:**
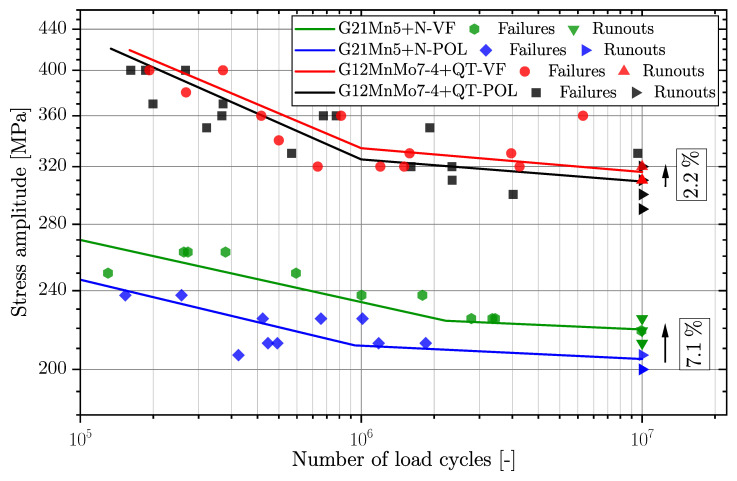
Fatigue test results of series G12MnMo7-4+QT and G21Mn5+N in vibratory finished and polished surface condition for load–stress ratio R=−1.

Figure 7 depicts the fatigue data for the cast steel series G21Mn5+N and G12MnMo7-4+QT for the polished and vibratory finished surface conditions. It can be clearly seen that the increase in fatigue strength is somewhat reduced compared to the aluminium alloy. One reason might be due to the increased strength of the material and therefore a reduced plastic deformation in the surface layer. The low-strength cast steel alloy revealed an increase in $\Delta \sigma_{LLF}$ by 7.1%, and the high-strength cast steel alloy just 2.2%. This confirms the previous assumption that the applied vibratory finishing process is no longer capable of increasing the fatigue strength significantly. It should be noted that the vibratory finishing treatment parameters and applied ceramics are identical for both cast steel materials; see Section 2. Both cast steel series revealed quite similar slopes $k_{1}$ in the finite life region.

Series G12MnMo7-4+QT exhibited a significant reduction of the fatigue scatter index, although series G21Mn5+N remains almost unaffected; see summarized fatigue data in Table 5. The aim of this experimental work was to build-up a fatigue test database on the effect of vibratory finishing versus manual polished surface conditions for cast aluminium and cast steel alloys. This database is further on used as experimental reference to extend the layer-based fatigue assessment model towards effective stress ratio effects.

### 3.2. Short and Long Crack Propagation

In order to facilitate a fracture-mechanical approach for fatigue assessment, short and long crack growth data are necessary for all investigated cast alloys. All crack growth data were evaluated at a probability of occurrence of $P_{Occ}=50\%$. Short and long crack growth behaviour of the aluminium series AlSi8Cu3-A and AlSi8Cu3-B, including detailed analysis of the microstructural relationship as well as a generalized dataset, is already reported in [93].

**Figure 8:**
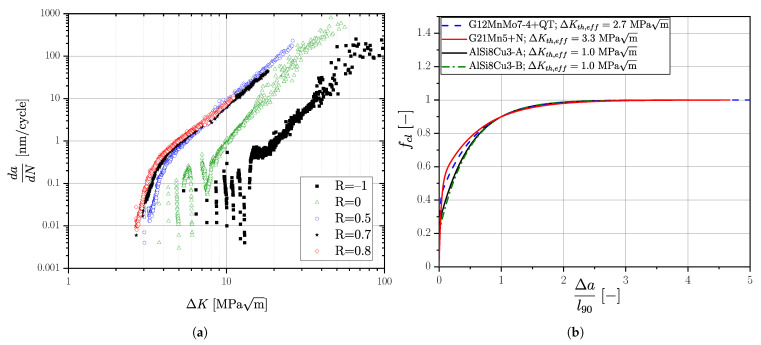
Crack growth data: (**a**) Short and long crack growth curves for series G12MnMo7-4+QT and (**b**) Generalized R-curves for all investigated cast alloy series.

Moreover, the crack growth behaviour of series G21Mn5+N was analyzed in detail by the authors in [136]. It should be mentioned, that additional experiments were conducted to extend previous studies for statistical improvement. The crack closure factor representing the generalized R-curves according to Kolitsch et al. [137] is summarized in Figure 8b for all investigated alloys. Crack growth data of series G12MnMo7-4+QT have been extended for different stress ratios within this work; compare to Figure 8a as reference. A summary of the R-curve data for this high strength cast alloy is given in Table 6. The long crack growth threshold values are given in Table 7.

### 3.3. Cyclic Elastic-Plastic Material Behaviour

**Figure 9:**
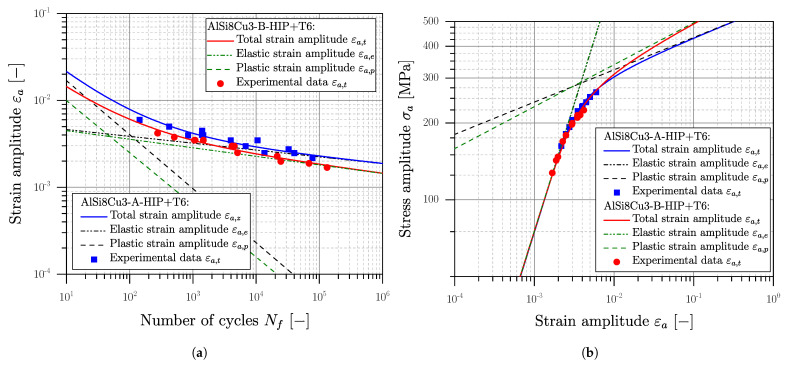
Low cycle fatigue results of AlSi8Cu3 cast alloy for $P_{Occ}=50\%$ (**a**) Manson–Coffin curve and (**b**) Stabilized cyclic stress–strain behaviour.

**Figure 10:**
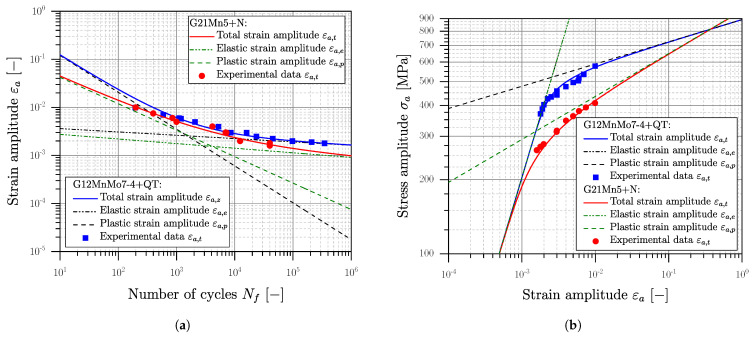
Low cycle fatigue results of cast steel alloys for $P_{Occ}=50\%$ (**a**) Manson–Coffin curve and (**b**) Stabilized cyclic stress–strain behaviour.

The statistical evaluation of the experimental results was conducted according the standard [116,122], defining the number of cycles to failure $N_{f}$ as a ten-percent decrease in the stabilized hysteresis. Subsequent, a strain-based low-cycle-fatigue study is conducted for all materials and evaluated according to Manson [138], Coffin [139] and Basquin [140] using the elastic–plastic strains at $N_{f}/2$. The evaluated curves are depicted in Figure 9 for the material AlSi8Cu3 as well as in Figure 10 for the steel alloys.

The total strain amplitude of the Manson–Coffin curve can be calculated as superposition of the elastic $\epsilon_{a,e}$ and plastic $\epsilon_{a,p}$ portion, represented by Equation (12) [138,139,140]. The elastic portion of the Manson–Coffin curve is given by the fatigue strength coefficient $\sigma_{f}^{\prime }$, the Young’s modulus $E^{\prime }$ and the fatigue strength exponent *b* of Basquin. For the plastic part in Equation (12), a ductility coefficient $\epsilon_{f}^{\prime }$ and the ductility exponent *c* are used [139,140,141].

(12) $$\epsilon_{a,t}=\epsilon_{a,e}+\epsilon_{a,p}=\frac{\sigma_{f}^{\prime }}{E^{\prime }}\cdot {\left(2N_{f}\right)}^{b}+\epsilon_{f}^{\prime }\cdot {\left(2N_{f}\right)}^{c}$$

The stabilized cyclic stress–strain curve, depicted in Figure 9b and Figure 10b, was evaluated according to the Ramberg–Osgood formulation [142], given in Equation (13).

(13) $$\epsilon_{a,t}=\epsilon_{a,e}+\epsilon_{a,p}=\frac{\sigma_{a}}{E^{\prime }}+{\left(\frac{\sigma_{a}}{K^{\prime }}\right)}^{\frac{1}{n^{\prime }}}$$

The cyclic hardening coefficient $K^{\prime }$ is calculated following Equation (14) utilizing the parameters of the Manson–Coffin curve as well as the cyclic strain hardening exponent $n^{\prime }$, as given in Equation (16) [143,144].

(14) $$K^{\prime }=\sigma_{f}^{\prime }\cdot {\left(\epsilon_{f}^{\prime }\right)}^{-n^{\prime }}$$

(15) $$n^{\prime }=\frac{b}{c}$$

Low cycle fatigue experiments were conducted for the HIP+T6 treated aluminium series AlSi8Cu3-A-HIP+T6 and AlSi8Cu3-B-HIP+T6 due to the quite brittle behaviour of the defect-afflicted T6 series. The cyclic stress–strain curves in Figure 9b are similar; the low cycle fatigue strength of position B is somewhat reduced due to the coarser microstructure; see Figure 9a. Comparing the 0.01% yield stress of YS=153 MPa of position A, and respectively YS=129 MPa for position B, a small fraction of cyclic hardening can be assumed. For both cast steel series cyclic softening can be perceived clearly, comparing the initial yield strength of LYS=323 and YS=549; see Figure 10b. A summary of the elastic–plastic fatigue experiments is given in Table 8.

To numerically simulate the redistribution of the residual stress state in the surface layer due to local work hardening, a proper cyclic plasticity material model has to be evaluated from the established experimental database. The superposition of notches and residual stresses in the surface layer enforces the simulation of local mean-stress relaxation by cyclic loading. The parameters of the combined hardening simulation model are explained in Section 1; see Equations (2)–(6). Two back stresses are considered for the non-linear kinematic part of the combined hardening model; see summary of the determined parameters in Table 9.

**Figure 11:**
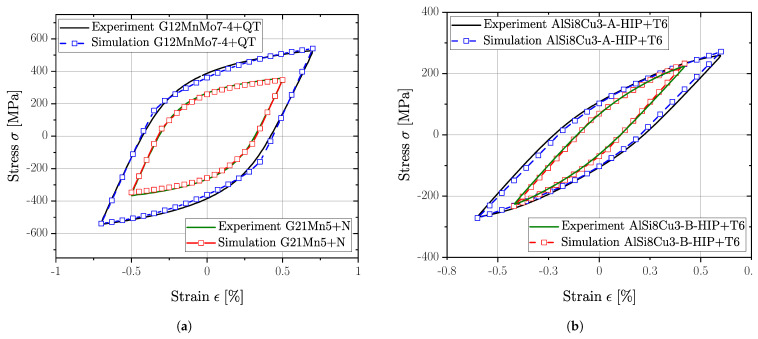
Validation of non-linear combined hardening models at stabilized condition for selected LCF-tests: (**a**) Cast steel alloys and (**b**) Cast aluminium alloys.

A non-linear finite element analysis was carried out using Simulia Abaqus^®^ CAE/2022 to validate the combined hardening model against experimental specimen test data. Therefore, single element tests were conducted to evaluate the stabilized hysteresis loop at selected strain amplitudes using axisymmetric elements of type CAX4R. Figure 11 depicts experimental and simulated stabilized hysteresis loops for all investigated series.

### 3.4. Fractography and Statistics of Extremes

**Figure 12:**
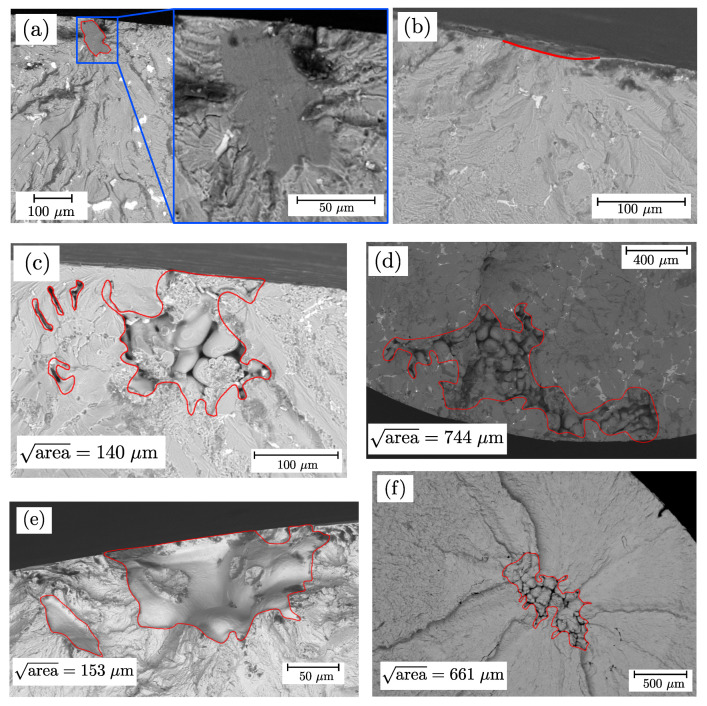
Main fracture initiating defects of the investigated specimen series for polished and vibratory finished surface condition: (**a**) Slip band and (**b**) Surface initiation; (**c**) Shrinkage AlSi8Cu3-A and (**d**) Shrinkage AlSi8Cu3-B; (**e**) Shrinkage G12MnMo7-4+QT and (**f**) Bulk shrinkage G21Mn5+N.

All fracture surfaces of the fatigue tested samples were analysed with regard to crack initiation site and failure mechanism. Selected fracture surfaces of tested samples are depicted in Figure 12, where the red line highlights the crack initiation spot. Specimen series AlSi8Cu3-A-HP+T6 exhibited different failure mechanisms like slip bands, oxide films, inclusions or small surface pits for both surface states. All these inhomogeneities were located near, or at, the specimen surface. Figure 12a displays a slip surface interacting with small oxide films, which can be identified as dark areas in the blue-framed sub-figure. A fracture surface with a crack initiating from a surface pit is depicted in Figure 12b. Porosity induced crack initiation is identified as the main failure mechanism for aluminium specimen series Alsi8Cu3-A-T6 and Alsi8Cu3-B-T6 for both surface finishing treatments; see Figure 12c,d, where position A revealed a mean defect size of ${\sqrt{\mathrm{area}}}_{Pocc=50\%}=101\;\mu m$ and position B ${\sqrt{\mathrm{area}}}_{Pocc=50\%}=406\;\mu m$. The distance of fracture initiating defects from the surface for vibratory finished samples is slightly increased compared to polished ones. One reason might be the compressive residual stress state in the surface layer [145], shifting the crack initiation site below the surface until an increasing effective load–stress ratio $R_{eff}$ is achieved again.

Figure 12e depicts porosity as the main failure mechanism of series G12MnMo7-4+QT for polished and vibratory finished surface, resulting a mean imperfection size of ${\sqrt{\mathrm{area}}}_{Pocc=50\%}=127\;\mu m$. Almost all fracture initiating defects are superficial. Regarding series G21Mn5+N, a larger fraction are bulk volume defects, as depicted in Figure 12f. The statistical mean of the projected imperfection size is ${\sqrt{\mathrm{area}}}_{Pocc=50\%}=279\;\mu m$. The evaluation of the projected area of the defects as well as the distance from the surface were measured using Fiji [146] as image processing tool. A spline was drawn manually around the contour of the defect, leading to smaller but more precisely defined defect sizes compared to Murakami’s convex hull approach. Moreover, a direct comparison of the projected area from computed tomography and fractography is therefore possible. Interacting defects were defined according to the proposal of Åman et al. [147], that states if the distance between two defects is less than the size of the smaller defect, both defects have to be considered as interacting. In this case, the sum of the projected $\sqrt{\mathrm{areas}}$ was taken into account.

**Figure 13:**
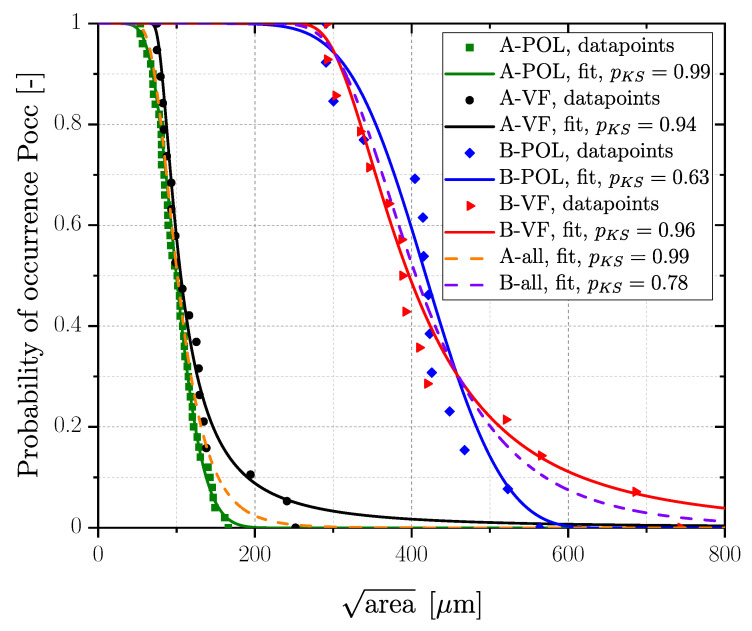
Defect distributions for AlSi8Cu3-A+T6 and AlSi8Cu3-B+T6.

Statistics of extreme were applied to study the distribution of fracture initiating defects, as introduced in Section 1. Regression analysis using Matlab^®^ 2020b parametrized the generalized extreme value (GEV) distribution functions for each series. The general distribution function is given in Equation (10). The parameters of the distribution function, namely, location μ, scale δ and shape ξ parameter were determined using the maximum likelihood estimation [148], followed by a Kolmogorov–Smirnov (KS) test at a significance level of 5% to proof the goodness of fit for the evaluated distribution [149]. Figure 13 depicts the cumulative GEV distributions for AlSi8Cu3-A-T6 and AlSi8Cu3-B-T6 series, both for polished and vibratory finished condition. The maximum sized defects of the vibratory finished condition are slightly increased compared to polished ones, but the mean value is quite similar for both specimen series. Series AlSi8Cu3-A-T6 results ${\sqrt{\mathrm{area}}}_{Pocc=50\%}=99.7\;\mu m$ for the polished and ${\sqrt{\mathrm{area}}}_{Pocc=50\%}=105\;\mu m$ for the vibratory finished state and series AlSi8Cu3-B-T6 ${\sqrt{\mathrm{area}}}_{Pocc=50\%}=418\;\mu m$ for polished and ${\sqrt{\mathrm{area}}}_{Pocc=50\%}=397\;\mu m$ for vibratory finished samples. Since the scatter bands of both series are overlapping for each surface state, a common GEV was evaluated summarizing the defects of polished and vibratory finished surface state for each specimen series; see dashed orange line in Figure 13 for series AlSi8Cu3-A-T6 and dashed purple line for series AlSi8Cu3-B-T6. Moreover, the basic defect distribution for polished and vibratory finished state within the whole sample is the same, because the difference is caused by the residual stress field in the surface layer.

**Figure 14:**
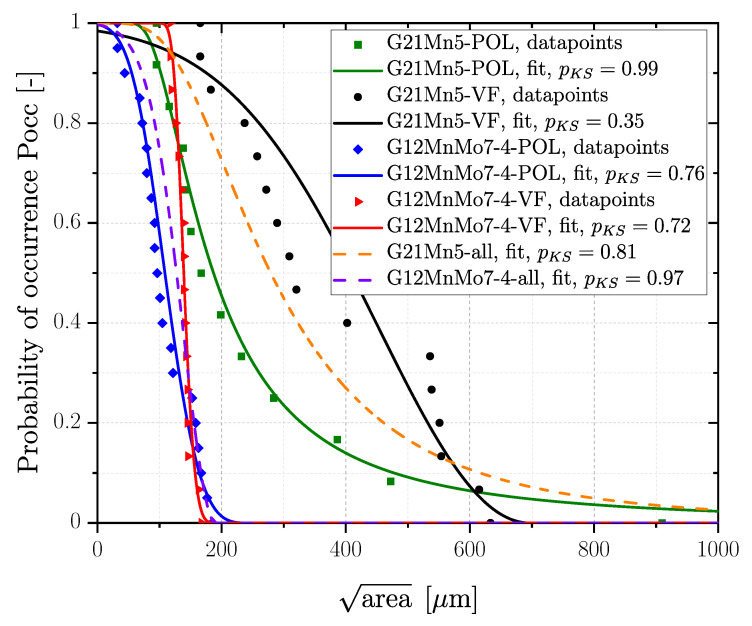
Defect distributions of G12MnMo7-4+QT and G21Mn5+N.

A summary of the evaluated distribution parameters for all defects for polished and vibratory finished state as well as the related projected ${\sqrt{\mathrm{area}}}_{Pocc=50\%}$ for all fracture initiating defects is given in Table 10. Fracture surfaces of steel samples were statistically analysed yielding the distributions plotted in Figure 14. Series G12MnMo7-4+QT reveals quite similar GEVs for the polished and vibratory finished surface; see red marked data for vibratory finished (${\sqrt{\mathrm{area}}}_{Pocc=50\%}=139\;\mu m$) and blue marked for polished (${\sqrt{\mathrm{area}}}_{Pocc=50\%}=109\;\mu m$) data in Figure 14. In addition, a combined GEV distribution for polished and vibratory finished was evaluated as well; see dashed purple line in Figure 14 and data summary in Table 10. Series G21Mn5+N reveals a comparably large scatter band of the assessed GEV for polished (${\sqrt{\mathrm{area}}}_{Pocc=50\%}=187\;\mu m$) and vibratory finished (${\sqrt{\mathrm{area}}}_{Pocc=50\%}=409\;\mu m$) surface condition. Concluding, a common GEV was evaluated as well for this cast material; see dashed orange line in Figure 14. It should be mentioned that the evaluated GEV distributions are based on measurement of the projected imperfection area of each fatigue tested specimen.

### 3.5. Computed Tomography

Computed tomography investigations were conducted only for the aluminium series, applying a threshold-based image post processing of the raw data, as detailed in a previous study of the authors [94]. A clustering of interacting porosity networks was incorporated following the proposal of Åman et al. [147]. Subsequently, the projected area was evaluated perpendicular to the load direction. As mentioned in the previous section, the region of failure initiation for cast aluminium alloys ranges from superficial defects up to bulk defects with a minimum distance to the surface of more than 500 μm. Thus, the computed tomography raw data have been evaluated for different layers. The size of the layer was set in accordance to the position of the majority of fracture-initiating pores. Hence, a layer starting from the surface up to a distance of 100 μm was selected for the polished specimens of series AlSi8Cu3-A-T6 and 250 μm for the vibratory finished ones based on the analysis of the fracture surfaces. Series AlSi8Cu3-B-T6 revealed a size for the surface layer of 400 μm for the polished surface and 500 μm in the case of a vibratory finished state.

**Figure 15:**
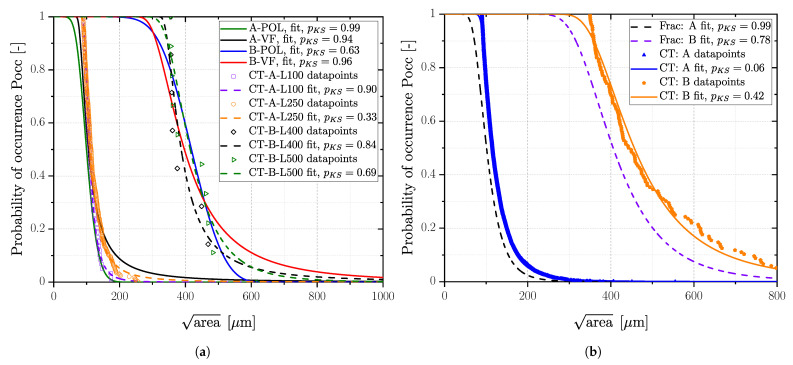
Comparison of fractography and computed tomography for AlSi8Cu3-A+T6 and AlSi8Cu3-B+T6: (**a**) Inspected layers for predominant region of failure in case of POL and VF samples and (**b**) Mean fractographic distribution compared to computed tomography.

The distribution of defects detected by CT using POT has asymptotic properties, thus a generalized Pareto distribution can be fitted to the data. Most of the pores are not critical regarding fatigue resistance, therefore a proper threshold value has to be chosen above which pores considered as fatigue critical. Romano et al. [108] determined the threshold value of CT-data using mean excess plots for additive manufactured parts. Mean residual life plots and threshold stability plots for the generalized Pareto distribution [150] were used to determine an applicable threshold set value for the defect size of the CT data of the cast aluminium parts [151,152]. Once a proper threshold is calculated, the mean residual life over the selected threshold value becomes linear [108]. Mean residual life plots and threshold choice plots revealed a threshold value of about 90 μm for series AlSi8Cu3-A-T6 and 350 μm for series AlSi8Cu3-B-T6, resulting in the GEV distributions depicted in Figure 15. In addition, a comparison of the CT-data and fractography for the fatigue critical layers was performed, leading to a sound accordance of the distributions; see Figure 15a.

For applications in practice, the size of the fatigue critical layer is not known in advance. One can choose the highly stressed volume (HSV) for CT scan as well, but the statistical size effect has to be considered accordingly [94]. Incorporating all defects of the specimens’ cross section results in distributions as depicted in Figure 15b. The mean values of the GEV for both Al-series as well as the associated parameters are given in Table 11. The distributions are shifted slightly to larger defects due to the effect of HSV.

### 3.6. Residual Stress Measurements

**Figure 16:**
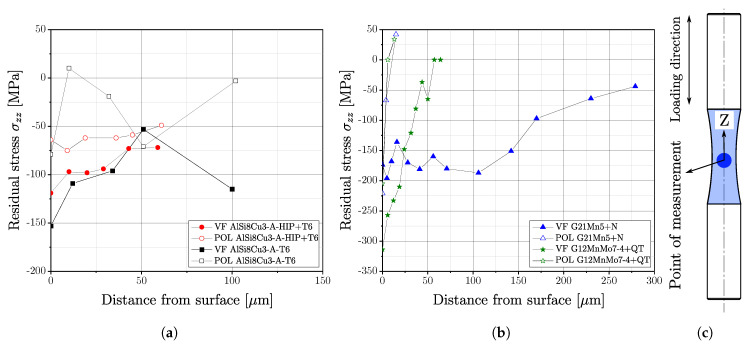
Residual stress measurements in the surface layer of polished (POL) and vibratory finished (VF) specimens: (**a**) Magnitude of residual stresses in z-direction for aluminium series, (**b**) Magnitude of residual stresses in z-direction for steel series, (**c**) Position of measurement for selected specimen geometry.

Residual stresses were measured for all investigated specimen series except AlSi8Cu3-B-T6 in several positions in the surface layer, depicted in Figure 16a,b. The residual stress state was measured in the test cross-section of the specimen along the loading direction, representing the z-axis in Figure 16c. It is clearly shown in Table 12 that machined and vibratory finished specimens are characterised by a compressive residual stress state on the surface, but at a certain distance from the surface, the residual stress state undergoes a rapid change. Figure 16a depicts the magnitude of the measured residual stress state in the surface layer for the aluminium series, whereas Figure 16b illustrates the residual stress profiles for the steel series.

Polished specimens of the cast steel series revealed a strong gradient towards tensile values since the initiated compressive residual stress state at the surface changes between 10 μm and 15 μm to tensile; see Figure 16b. However, apart from the initial surface condition, the course of residual stresses is quite similar for the polished steel series. In contrast, the vibratory finished condition revealed different distributions of the stress state; see Figure 16b. Whereas series G12MnMo7-4+QT still possess a large gradient with transition into tensile stress state at a depth of about 60 μm, series G21Mn5+N exhibits a decreased gradient and therefore compressive residual stresses up to a depth of 300 μm. One reason might be the comparably high strength and increased hardness of the quenched and tempered series G12MnMo7-4. Thus, reducing the impact of the ceramic particles of vibratory finishing process featuring small plastic deformations at the surface layer, whereas the more ductile series G21Mn5+N is affected a somewhat due to its lower yield limit.

The aluminium series AlSi8Cu3-A-T6 indicates the same behaviour as the steel parts; the compressive layer of the polished surface is quite small, and the gradient of the vibratory finished surface is decreased; see Figure 16a. In contrast, series AlSi8Cu3-A-HIP+T6 features a low gradient for the polished state as well. One reason for this still-decreased gradient of polished surface condition might be a pre-existing residual stress state resulting from the HIP process, apparent when comparing series AlSi8Cu3-A-T6-POL and AlSi8Cu3-A-HIP+T6-POL in Figure 16a.

### 3.7. Surface Layer Hardness

**Figure 17:**
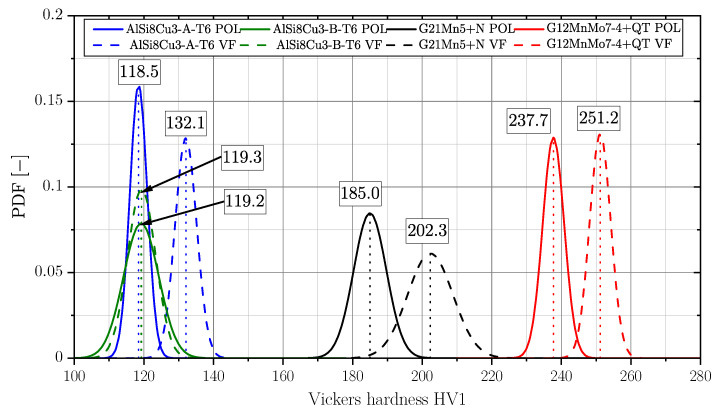
Surface hardness HV1 of series AlSi8Cu3-A-T6, AlSi8Cu3-B-T6, G21Mn5+N and G12MnMo7-4+QT for polished and vibratory finished surface. Boxed values represent the mean values of the normal distributions.

Vickers hardness measurements were conducted at the investigated specimens’ surfaces. Comparing the ratio of the smooth roughness profile for both surface conditions to the indentation depth, the influence of the surface profile on hardness can be neglected. Figure 17 depicts the probability density functions (PDF) of the Vickers Hardness (HV1) and the corresponding mean value in the framed box as well. Except for series AlSi8Cu3-B-T6, all investigated samples revealed an increase in surface hardness after vibratory finishing. Depending on the strength, respectively hardness of the material, enhancement ranges between 5.7% for the high strength cast steel alloy up to 11.5% for the aluminium alloy of position A. The lower strength cast steel alloy reveals an increase of 9.4%. Only series AlSi8Cu3-B-T6 revealed no significant change of hardness, but the scatter index of the polished specimens is somewhat increased. One reason might be coarse microstructure and high degree of porosity of these samples series implying a reduced plastic deformability.

**Figure 18:**
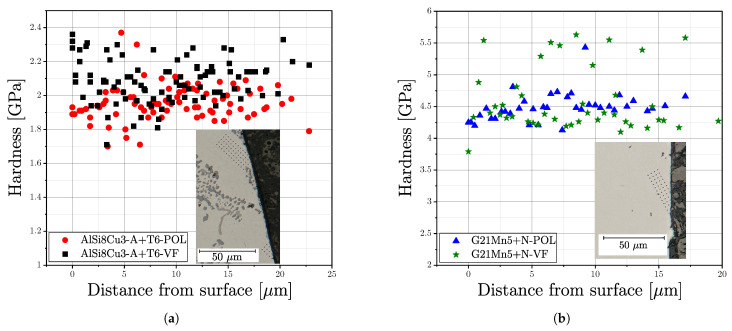
Nanoindentation measurements of series (**a**) AlSi8Cu3-A-T6 and (**b**) G21Mn5+N.

In addition, nanoindentation experiments were carried out in the cross section of the surface layer in order to obtain information about the hardness gradient by polishing or vibratory finishing. It should be mentioned that the indenter tips of Vickers for macrohardness and Berkovich for nanoindentation are different and the hardness results are therefore not directly comparable. But the main focus is laid on the course of the hardness in the surface layer, dependent on the material series and surface treatment condition. Figure 18 depicts the obtained results for series AlSi8Cu3-A-T6 and G21Mn5+N. A grid of indentation points was positioned at the surface layer, exemplary shown in the subfigures of the plots in Figure 18. In case of series AlSi8Cu3-A-T6, a slight increase in hardness can be noticed comparing the red and black datapoints at the surface position in Figure 18a. In contrast, the cast steel alloy G21Mn5+N reveals no recognizable change of hardness in the surface layer; see Figure 18b.

However, the intensity of the vibratory finishing process is quite small for the used ceramic and plastic process media in case of steel samples, in contrast to literature data, where increased hardness values could be observed using special finishing media [54,55]. The scatter of the datapoints can be justified by the effect of the local microstructure within the comparably low indentation depth of only 150 nm.

## 4. Cyclic Stability of Residual Stresses

It was shown in Section 3 that the fatigue behaviour of the investigated materials changes quite significantly towards increased fatigue performance by vibratory finishing, with exception of the high strength cast steel alloy G12MnMo7-4+QT, lacking a pronounced increase in similar strength. Manufacturing process based defects such as shrinkage porosity for example are playing a major role in terms of fatigue performance; see Section 1. Depending on size, shape and position of inhomogeneities in respect to the surface, varying fatigue strength values can be obtained. In the vicinity of each defect, the residual stress field within the appropriate surface layer may become modified due to cyclic hardening or softening. Sausto et al. [24] proposed to determine the residual stress relaxation using elastic–plastic FE simulations to predict the evolution of the residual stress state in the surface layer for additive manufactured samples with different surface layer treatments. As depicted in the experimental work, the effect of vibratory finishing versus manual polishing depends on the locally applicable residual stress ratio for each material, as the defect distribution itself is not changed by the surface treatment process itself. Thus, the key part of this work focusses on the extension of layer-based fatigue assessment tool, as previously introduced for cast aluminium alloys [94], towards consideration of the effective residual stress state for each extremal defect. This section deals with the predicted stability of the residual stress state in the surface layer during fatigue loading by simulation.

### 4.1. Approximation of Residual Stress Profile

In order to perform a numerical simulation on residual stress relaxation, a continuous stress profile into the specimen depth is essential. For fatigue analysis, only the first material layers are of particular importance. Therefore, the residual stresses of the tested specimens were measured to a depth of approximately in the size of the El-Haddad length $a_{0}$. These residual stresses must be self-equilibrated within the considered part. According to the findings of Smith et al. [153], the residual stress field within a cylindrical body must satisfy the equilibrium relationships stated in Equation (16).

(16) $$\int_{0}^{R_{cyl}}r\cdot \sigma_{zz}^{res}\left(r\right)dr=0$$

$R_{cyl}$ represents the maximum radius of the cylindrical specimen cross section and $\sigma_{zz}^{res}\left(r\right)$ is the axial stress function along the radius *r*. The initial function of $\sigma_{zz}^{res}\left(r\right)$ was evaluated based on the experimental measurements in the surface layer; values of the residual stresses in the material depth were calculated in an iterative process to fulfil Equation (16).

**Figure 19:**
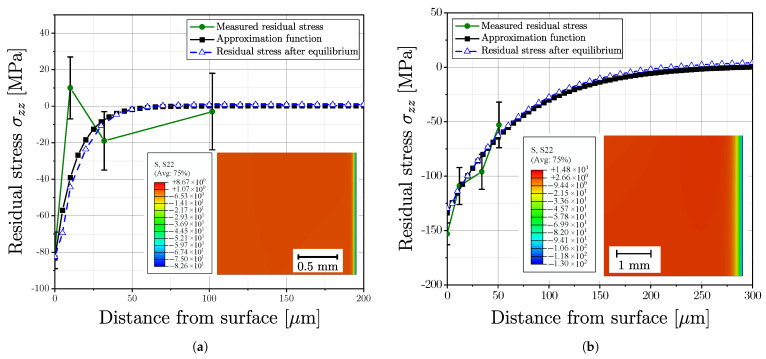
Approximated residual stress profile of series AlSi8Cu3-A-T6 and profile after equilibrium step in FE simulation for (**a**) Polished surface and (**b**) Vibratory finished surface.

**Figure 20:**
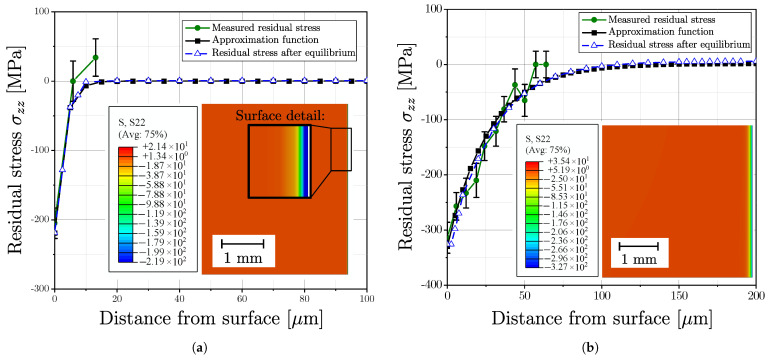
Approximated residual stress profile of series G12MnMo7-4+QT and profile after equilibrium step in FE simulation for (**a**) Polished surface and (**b**) Vibratory finished surface.

Figure 19 exemplary depicts the approximated residual stress profile for one aluminium series, (a) for polished surface and (b) for vibratory finished surface and Figure 20 for one cast steel alloy. The measurements for both surface post-treatment states revealed a high scatter of the derived residual stresses; see green line and related error bars at the measurement points. This scattering of residual stress data points can be attributed to the effect of local microstructure. An estimate of the approximation function was made as multiparametric exponential function. In the following step, the stress values of the approximation function were implemented in the FE-model as initial stress state, followed by an subsequent equilibrium step. The respective stress distribution in equilibrium state of the FE simulation is given in the sub plots and the blue lines as well.

### 4.2. Numerical Analyses

**Figure 21:**
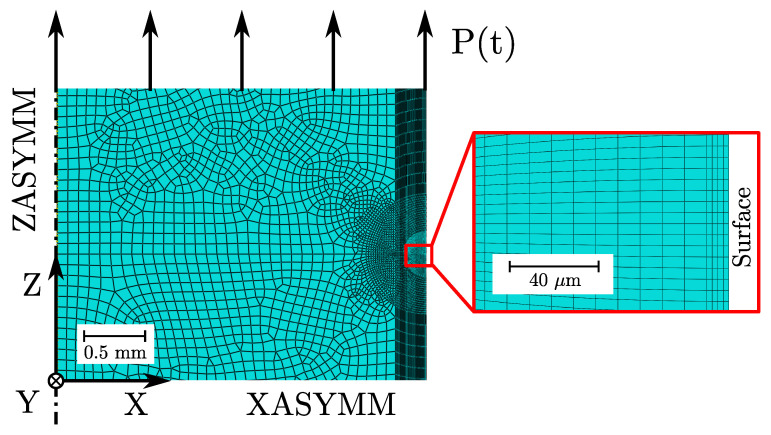
Simulation set-up to determine the stable effective load–stress ratio in the surface layer.

An axisymmetric model of the gauge section was used to investigate the cyclic evolution of the residual stresses inside the surface layer to reduce computational effort of the elastic–plastic FE-simulations in Abaqus CAE/2022. A user defined Python script was developed to set-up a proper axisymmetric model and subsequent postprocessing. A cyclic load function p(t) was defined for the applied surface until a stable stress–strain hysteresis is reached. The set-values for the combined hardening material model were taken from Table 9. Linear axisymmetric elements of type CAX4R were adopted to mesh the model. A very high mesh refinement was necessary to achieve numerical convergence concerning the steep stress gradient in the surface layer, especially for the polished samples. Hence, an element size starting with 2.5 μm was applied for local mesh seed. Thus, up to 18,000 axisymmetric elements were necessary to properly simulate the residual stress gradient in the surface layer; see red bounded box in Figure 21. XASYMM and ZASYMM boundary conditions were applied, depicted in Figure 21.

(17) $$R_{eff}=\frac{\sigma_{min}}{\sigma_{max}}=\frac{\sigma_{res,eff}-\sigma_{a}}{\sigma_{res,eff}+\sigma_{a}}$$

**Figure 22:**
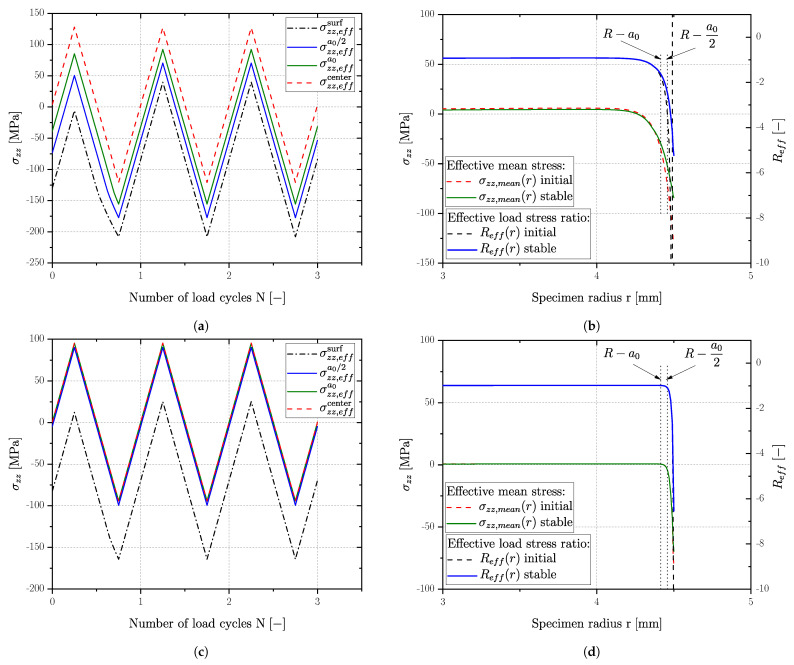
Simulation of cyclic residual stress behaviour in the surface layer, exemplifying effective mean stress course and stress ratio in AlSi8Cu3-A-T6 samples as (**a**) Stress history by VF (**b**) Effective stress ratio by VF (**c**) Stress history by POL (**d**) Effective stress ratio by POL.

Within post processing, stresses and strains were evaluated on user defined stress paths as a function of time or load cycles respectively and radial position in the specimen cross section. Figure 22a depicts the effective load stress for a vibratory finished sample on the surface $\sigma_{zz,eff}^{\mathrm{surf}}$, in a distance of $a_{0}/2$ from the surface $\sigma_{zz,eff}^{a_{0}/2}$, a distance of $a_{0}$ labelled as $\sigma_{zz,eff}^{a_{0}}$ and the applied load stress in the center of the specimen $\sigma_{zz,eff}^{\mathrm{center}}$. The effective stress state over the specimens’ radius *R* as well as the corresponding effective load–stress ratio are plotted in Figure 22b for the vibratory finished surface state. Similarly, Figure 22c,d depict the results for the polished surface condition of series AlSi8Cu3-A-T6. Calculation of the effective load–stress ratio $R_{eff}$ is conducted invoking Equation (17) for three different depth values in Table 13.

It is clearly evident in Figure 22a,b that plastic deformation in the surface layer takes place mostly in the first stress-controlled load cycle. Whereas the polished state reveals a plastic deformation only in the first few microns in the surface layer (see stress distribution in Figure 22d) vibratory finished samples revealed a larger zone of plastic influence up to a distance of $a_{0}$ from the surface.

Table 13 presents a summary of the stabilized effective load–stress ratio $R_{eff}$ of all investigated series in polished and vibratory finished condition. The values are given at the aforementioned distances from the surface ($a_{0}$, $a_{0}/2$) and at the surface. A positive value of the effective load–stress ratio $R_{eff}$ occurs if the residual stress state is somewhat higher than the applied load stress $\sigma_{LLF}$. All specimen series revealed a compressive stable effective load–stress ratio at the surface. In a distance of $a_{0}/2$ from the surface, $R_{eff}$ was compressive as well for the vibratory finished samples, whereas the polished samples approach the global applied load–stress ratio, except series AlSi8Cu3-A-HIP+T6 possessing a comparably flat gradient. At a distance of $a_{0}$ almost all $R_{eff}$ values are set to the global applied load–stress ratio, even though an effect of the shallower residual stress gradient remained.

**Figure 23:**
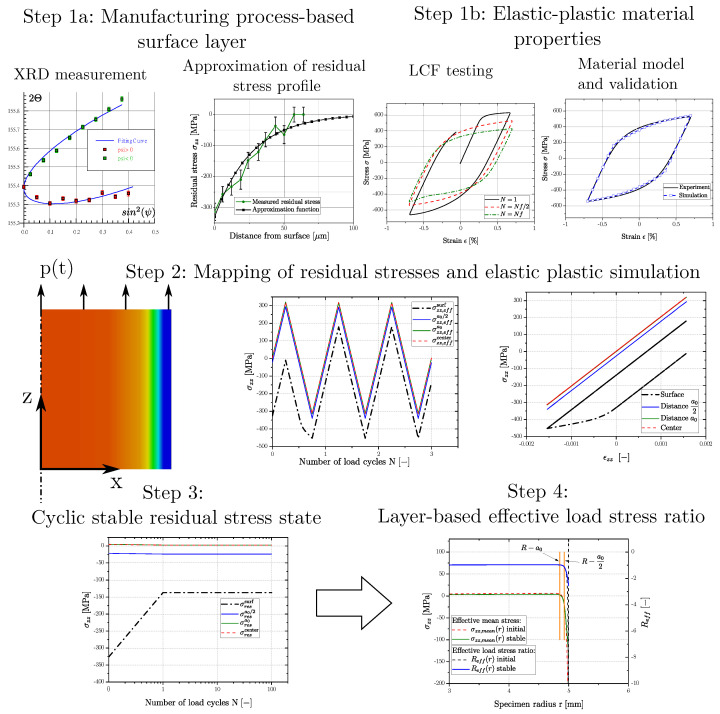
Schematic description of the process for determining the cyclic stable residual stress profile and effective load–stress ratio, which serve as input parameters for fatigue strength assessment.

The applied scheme for modelling the effective stable load–stress ratio of a sample subjected to cyclic loading is given in Figure 23. Step 1a represents the experimental part regarding residual stress measurement in the manufacturing process based surface layer. In parallel, a proper material model must be set-up for work hardening simulation as Step 1b for example by evaluating low cycle fatigue tests. Step 2 represents the conduction of the elastic plastic simulation of the updated residual stress profile in the surface layer. Finally, a stable residual stress state can be estimated along the specimens’ radius, Step 3, resulting the effective load–stress ratio as input parameter for subsequent fatigue assessment as Step 4.

### 4.3. Experimental Validation of Stabilized Hysteresis Loop

Although the validation of the invoked material model was realized by strain controlled single element tests, an experimental validation of the cyclic stable residual stress state on the surface was conducted as well for series G21Mn5+N up to long life fatigue limit. Thereby, the high cycle fatigue test was interrupted at selected numbers of load cycles to measure the surface residual stress state. The applied load stress amplitude is about 200 MPa, ensuring fatigue testing in the long life region.

**Figure 24:**
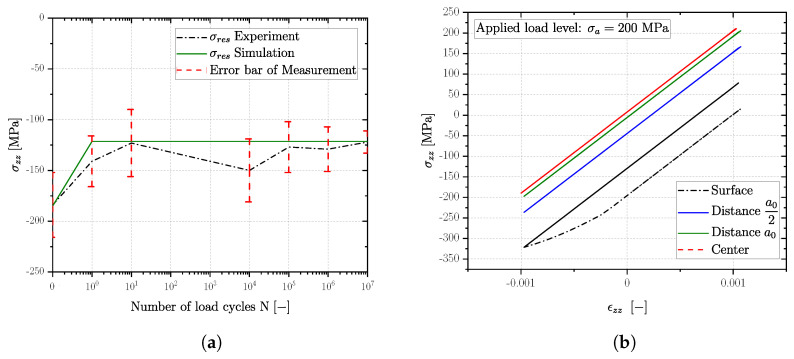
Validation of residual stress redistribution for series G21Mn5+N with vibratory finishing: (**a**) Comparison of simulation and experiment and (**b**) Cyclic stress strain behaviour in selected layers.

The course of the residual stress profile in dependency of the load cycle number is given in Figure 24a. It is clearly evident from the experimental results (black dash-dotted line with red scatter of the measurement) that the first cycles are most important regarding residual stress redistribution, achieving the stable residual stress in the first few load cycles. The observed increase in the compressive residual stress state on the surface at $10^{4}$ cycles my be due to deviation of the measurement set point. But the simulation is still within the measured scatter band. Overall, simulated residual stresses revealed a satisfying compliance to the measured mean values, evident in Figure 24a that the simulation always remains within the scatter band of the measurements. Moreover, a stable residual stress state is accomplished in the simulation within the first load cycle (see Figure 24b) due to plastic deformation during pressure loading.

## 5. Fatigue Model and Discussion

This section provides the implementation of the numerically simulated effective stable residual stress ratio into established fatigue models capable for defect tolerant design. The defect distribution is evaluated for each layer, and the obtained extremal defect size links to the fatigue threshold limit as Kitagawa–Takahashi diagram [26]. Otherwise, Murakami’s concept relates the projected extremal defect area of the assessed layer to the fatigue stress limit [21]. As pointed out in the previous section, the effect of effective residual stress ratio, and related change in micro hardness, within the evaluated layer shall be additionally considered to improve these fatigue design concepts further. Without the proper knowledge of the effective stress ratio, one cannot distinguish between manually polished and vibratory finished surface condition. Hence, the extended fatigue models shall be able to incorporate the effect of post surface finishing method properly.

The calculated fatigue limits are compared to the endurance value of the S/N-curves as given in Section 3. Table 4 and Table 5 depict the experimentally obtained long-life fatigue strengths of cast aluminum and cast steel as reference. The evaluated design strength of each series is based on a probability of fifty percent of the imperfection distribution. In case of aluminum alloys, the aggregated distribution of all CT-scanned samples has been utilized, compare to $P_{Occ}=50\%$ of AlSi8Cu3-A+T6 and AlSi8Cu3-B+T6 in Figure 15 of the highly-stressed volumes. In case of cast steel, the mean occurrence value of the defects is based on the fractographically determined distribution for G21Mn5+N and G12MnMo7-4+QT.

### 5.1. Kitagawa–Takahashi Diagram

**Figure 25:**
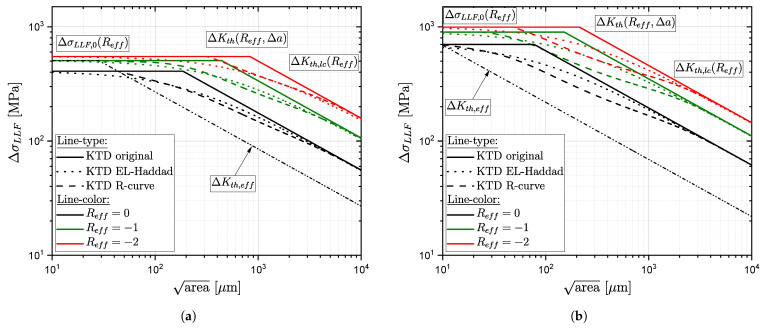
Schematic representation of the original Kitagawa diagram, as well as its modifications for three different effective load–stress ratios ($R_{eff}$): (**a**) G21Mn5+N and (**b**) G12MnMo7-4+QT.

The Kitagawa–Takahashi diagram (KTD) is one of the most widely used fatigue strength models based on fracture mechanics, which considers manufacturing process induced material inhomogeneities. In this work, El-Haddad’s extension et al. [61] and the effect of crack closure mechanisms according to Chapetti [91] are utilized for fatigue strength assessment. The fracture mechanical crack closure integration was already extended regarding defect distribution and layer based fatigue assessment for AlSi-cast parts in [94,135]. It is denoted in this work as R-curve concept; see Equation (18). Figure 25 depicts the KTD in original formulation (solid lines), with the modification by El-Haddad (dotted line) and the R-curve concept (dashed line) for different effective load–stress ratios $R_{eff}$ for cast steel alloys G21Mn5+N and G12MnMo7-4+QT. The illustrated range of the cyclic stable effective load–stress ratio is taken in accordance to the simulation study, results summarized in Table 13, as shown in detail in Section 4, for the respective series and surface state.

The upper border $\Delta \sigma_{LLF,0}\left(R_{eff}\right)$ represents the fatigue strength of the defect free base material. It can be calculated from the Ramberg–Osgood parameters of the cyclic stable stress strain curve, or experimentally as S/N-curve from defect-free samples. For the aluminium alloy, $\Delta \sigma_{LLF,0}\left(R=-1\right)$ was obtained from fatigue testing of HIP+T6 treated samples. Thus, the local base material strength for the effective local load–stress ratio $\Delta \sigma_{LLF,0}\left(R_{eff}\right)$ can be calculated using the Haigh diagram in accordance with the standard [58]. For both cast steel alloys, the base material strength was estimated using the Ramberg–Osgood parameters according Equation (9), as no defect free samples were available for fatigue testing. The effective load–stress ratio was taken into account in the same way as for the aluminium alloy.

(18) $$\Delta \sigma_{LLF}\left(\sqrt{\mathrm{area}},R_{eff}\right)=\frac{\Delta K_{th,\Delta a}\left(R_{eff}\right)}{Y\cdot \sqrt{\pi \cdot ({\sqrt{\mathrm{area}}}_{P_{Occ}=50\%}-a_{0,eff})}}$$

**Figure 26:**
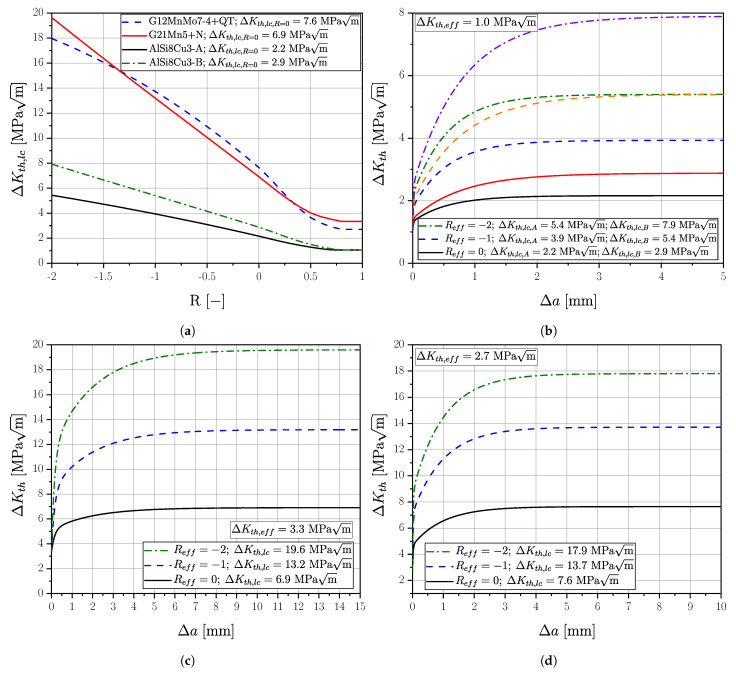
Crack growth data for the investigated materials illustrating the effective stress ratio $R_{eff}$: (**a**) Newman curves and (**b**) R-curve of material AlSi8Cu3 (**c**) R-curve of material G21Mn5+N and (**d**) R-curve of material G12MnMo7-4+QT.

The long crack threshold value $\Delta K_{th,lc}\left(R_{eff}\right)$ as function of the effective load–stress ratio can be determined from the Newman curve [103] for the respective material; see Figure 26a and Equation (20), representing the right border of the KT-diagram. The transition between crack threshold and base material strength is characterized by the build up of extrinsic shielding mechanisms, which can be represented by the cyclic R-curve, as given in Figure 26b for aluminium alloy AlSi8Cu3-AB, in Figure 26c for cast steel G21Mn5+N, and in Figure 26d for cast steel G12MnMo7-4+QT. Depending on the effective load–stress ratio $R_{eff}$, the local crack threshold affected by build up of closure mechanisms is a function of the crack extension, as proposed by Maierhofer et al. [92]. The course of the stress-intensity-factor-range crack-length dependency is calculated using Equation (8) in Figure 26b–d. Summing up, the effective stress ratio $R_{eff}$ affects the fracture mechanical long-crack threshold values quite severely. The difference decreases for short cracks, and becomes zero in case of the effective stress intensity factor $\Delta K_{th,eff}$ implying no crack elongation Δa. The R-curve concept is capable of considering this complex dependency, El-Haddad’s more simpler KT-modification takes only the long-crack dependency into account.

The effect of defect location within the evaluated layer, e.g., close to the surface or within bulk volume, is considered by the geometry factor *Y*. It is set to 0.73 for superficial and 0.65 for internal crack initiating imperfections, according to Radaj [89]. The depicted S/N-curves representing the mean value of fatigue testing implying both locations of fracture; see Section 3. Hence, a mean geometry correction factor was calculated for each specimen series based on the fractographic investigations, resulting probabilistic mean values between 0.65 and 0.73.

(19) $$\Delta \sigma_{LLF}\left(\sqrt{\mathrm{area}},R_{eff}\right)=\frac{\Delta K_{th,lc}\left(R_{eff}\right)}{Y\cdot \sqrt{\pi \cdot ({\sqrt{\mathrm{area}}}_{P_{Occ}=50\%}+a_{0})}}$$

Finally, the fatigue strength for a probability of occurrence of 50% can be evaluated as intersection of defects’ size and the respective limit line (R-curve or El-Haddad) in the KT-diagram. The size of fracture initiating defects ${\sqrt{\mathrm{area}}}_{P_{Occ}=50\%}$ is based on the GEV-distributions of each series; see results of statistical analysis of defects in Section 3. As the basic defect distribution in the highly stressed material volume is the same, the observed difference in fatigue strength between manually polished and vibratory finished samples is traced back to the effect of the effective residual stress state. The stress ratio *R* affects the long-crack threshold and thereby the design limit curve of the KTD. Equation (20) depicts the dependency of the long-crack threshold range on the stress ratio, parametrized for each material as illustrated in Figure 26. Table 14 summarizes the calculated KTD fatigue design strengths for each cast alloy assuming that the load–stress ratio R=−1 is applicable. This neglects purposely the effective load–stress ratio in the surface layer. Thus, this simplification can not distinct between manual polishing and vibratory finished surface treatment. The defect distribution is properly considered as $\sqrt{\mathrm{area}}$-value at an occurrence level of fifty percent for each series.

(20) $$\Delta K_{th,lc}\left(R\right)=\Delta K_{th,lc,R0}{\left(\frac{1-\gamma }{\left(1-A_{0}\right)\left(1-R\right)}\right)}^{-(1+C_{th}\cdot R)}$$

**Figure 27:**
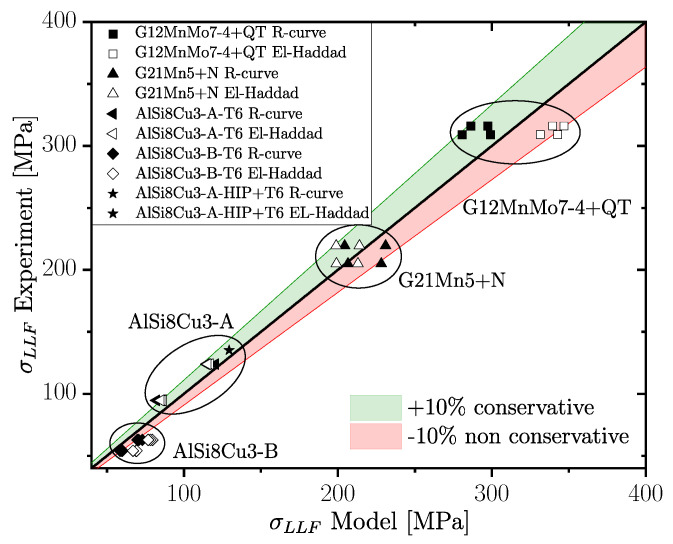
Comparison of experimental data and model predictions for fatigue assessment using the layer-based probabilistic Kitagawa diagram with $R_{eff}$ extension.

To incorporate the effective stress ratio within the surface layer for fatigue assessment, one has to be replace *R* by $R_{eff}$ in Equation (20). This lead to a sound match between experiment and calculation long-life-fatigue strength for the four investigated series, as depicted in Figure 27. Even though not pronounced distinctive for all cast alloys, the R-curve concept is preferable in conservative fatigue design compared to the simpler El-Haddad’s method. This holds especially true for the high-strength cast steel. Table 15 depicts the calculated fatigue strength of each surface treatment for the four different investigated cast alloys. In general, the KTD evaluation method with effective stress ratio extension is capable to reproduce the fatigue strength changes for polished and vibratory surface treatment satisfyingly. The observed improvement is different for each material, and is based on the effective stress ratio value within the surface layer for each material. Fatigue assessment based on the KT-diagram with effective stress ratio $R_{eff}$ leads to match between experiment and calculated fatigue life within ten percent in stress range for all investigated cast alloys and surface post-treatment methods.

It should be noted that the defect-free base material strength of each alloy is not obtained directly by fatigue testing, as the effective stress ratio effect has to be considered as well. In detail, the experimentally determined, corrected fatigue strength of the polished series AlSi8Cu3-A-HIP+T6 was used as base material strength in the assessment methodology for the aluminium series. Vibratory finished samples of this defect free series revealed a estimated fatigue strength of $\sigma_{LLF,50\%}=129.4$ MPa, which is 4.2% conservative compared to the experimental results for both, the R-curve as well as the EL-Haddad concept. The comparison to the experimental data are additionally depicted as black filled stars in Figure 27.

### 5.2. Murakami Concept

In order to incorporate the effect of local hardness $HV_{local}$ change and the effect of the local stress ratio $R_{eff}$, Murakami’s approach [21] is used as a second fatigue assessment method; see Equation (21). Again, the effective stress ratio $R_{eff}$ is put into operation instead of the load–stress ratio R. In addition, the measured change in surface hardness is considered for each surface treatment. The constants $C_{1}$ and $C_{2}$ of the extended Murakami approach for light metals by Noguchi et al. [109] were set to $C_{1}=1.43$ for surface, and $C_{1}=1.56$ for bulk defects, as well as $C_{2}=120$. The used local hardness values are referred in Figure 17 and detailed in Section 3. The effective local load–stress ratio was again taken from the elastic plastic simulations in stabilized condition, listed in Table 13.

(21) $$\sigma_{LLF}\left(\sqrt{area},R\right)\;=\;C_{1}\cdot \left(\frac{HV_{local}+C_{2}\cdot \frac{E_{Al}}{E_{St}}}{{\left(\sqrt{area}\right)}^{\left(1/6\right)}}\right)\cdot {\left[\frac{1-R}{2}\right]}^{\alpha }$$

**Figure 28:**
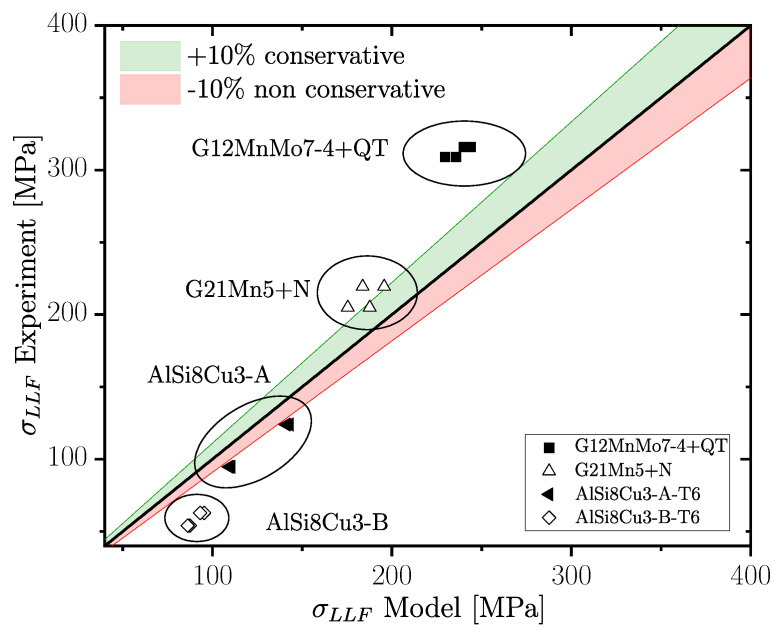
Comparison between experimental data and model predictions for fatigue assessment, considering local effective stress ratio $R_{eff}$ and hardness, using the modified Murakami approach.

Figure 28 depicts the differences between experimentally determined fatigue strength and calculated one for each cast alloy, using the modified Murakami approach with effective stress ratio $R_{eff}$. It is obvious that Murakami’s empirical approach is not as well applicable as the more sophisticated R-curve concept. But for more ductile materials, as in case of cast steel G12Mn5+N and the less porosity afflicted aluminium alloy charge AlSi8Cu3-A, Murakami’s approach is also quite close to the experimental reference. Table 16 summarizes the calculated fatigue strength values of Murakami’s method using the effective stress ratio $R_{eff}$ within the surface layer. It should be mentioned that the proposed extension by consideration of the effective surface layer stress ratio $R_{eff}$ generally improves the accuracy of Murakami’s method, using only the load–stress ratio *R*. But the observed deviation in calculated fatigue strength are between 26% conservative and up to 62% non-conservative, depending on the investigated material.

Summing up, it is clearly evident from the analysis of the results in this study, that the residual stress state has to be taken into account to distinguish between polished and vibratory finished surfaces for a proper estimation of the fatigue strength. Especially if fatigue samples are surface post-treated by vibratory finishing processes for ensuring sample manufacturing process reproducibility, the tested fatigue strength can be overestimated. But if the course of the effective stress ratio is considered within the surface layer, the fatigue assessment can be properly applied, as illustrated for cast alloys using the R-curve KTD method with $R_{eff}$ extension. It should be noted that the surface treatment effect can be assessed in a similar manner for materials without imperfections, in this case taking only the change of $R_{eff}$ by elasto-plastic simulations accordingly into account.

## 6. Conclusions

The effect of vibratory finishing and the associated residual stresses were taken into account for evaluation of fatigue performance of two cast steel alloys as well as one aluminium alloy with different microstructure and heat treatment conditions. To investigate the evolution of residual stresses during cyclic loading, comprehensive numerical analysis were performed incorporating the cyclic elasto-plastic material behaviour for each cast alloy. The obtained numerical results yield an effective stress ratio dependency for each layer and implemented in fracture mechanical fatigue models such as the Kitagawa–Takahashi diagram as effective stress ratio $R_{eff}$. In addition, the suitability of Murakami’s method was studied as well. Finally, the following conclusions can be drawn from the obtained results focussing on the effect of surface treatment and related effective stress ratio in fatigue design:

- Vibratory finishing has a significant impact on the high cycle fatigue strength and reduces the scatter index of fatigue test data compared to manually operated polishing processes.
- The impact of vibratory finishing on the surface layer depends quite strongly on the yield strength of the investigated material.
- For aluminium alloys, the benefit in fatigue strength can approach thirty percent, but for high-strength cast alloys the gained increase reduces to about three percent.
- A compressive residual stress state is achieved for all examined vibratory finished specimen series and results in a corresponding improvement of fatigue strength.
- Although global elastic loading is applied in fatigue testing, the residual stress profile in the surface layer is rearranged due to cyclic loading. The modification of residual stress state was investigated with a series of elastic–plastic finite-element simulations.
- The proposed methodology for incorporating the cyclic stable effective residual stress state $R_{eff}$ is capable for fatigue design of post-processing treatments such as vibratory finishing, but can be similarly applied to study other surface treatment processes such as shot-peening or case hardening as well.
- Quite accurate fatigue strength calculations within ten percent stress range deviations were obtained considering the cyclic stable residual stress profile of the numerical simulations at a certain distance from the surface using a probabilistic Kitagawa–Takahashi diagram including short crack effect, load–stress ratio and microstructure dependency.
- The more easily applicable approach of Murakami considering the local stable effective load–stress ratio and surface hardness leads to an increased fatigue scatter band, but is still applicable for ductile cast alloys with about fifteen percent in stress range deviation.

## Figures and Tables

**Table 1:** Nominal chemical composition of the investigated cast alloy AlSi8Cu3+T6 in weight percent.

Alloy	Si [%]	Cu [%]	Fe [%]	Mn [%]	Mg [%]	Ti [%]	Al [%]
AlSi8Cu3 [111]	7.5–8.5	2.0–3.5	0.8	0.15–0.65	0.05–0.55	0.25	balance

**Table 2:** Chemical composition in weight percent of the investigated cast steel alloys G12MnMo7-4 and G21Mn5.

Alloy	C [%]	Mn [%]	Mo [%]	Si [%]	Cr [%]	P [%]	S [%]
G21Mn5 [113]	0.17–0.23	1.1–1.3	-	max. 0.6	max. 0.3	max. 0.02	max. 0.015
G12MnMo7-4 [114]	0.08–0.15	1.5–1.8	0.3–0.4	max. 0.6	max. 0.2	max. 0.02	max. 0.015

**Table 3:** Overview of the fatigue tests performed on polished and vibratory finished samples for the three investigated materials.

Series’ Code	Surface Treatment [−]	Heat Treatment [−]	Load Ratio [−]	Number of Tests [−]
AlSi8Cu3-A-HIP+T6-POL	POL	HIP+T6	−1	56
AlSi8Cu3-A-HIP+T6-VF	VF	HIP+T6	−1	24
AlSi8Cu3-A-T6-POL	POL	T6	−1	51
AlSi8Cu3-A-T6-VF	VF	T6	−1	15
AlSi8Cu3-B-T6-POL	POL	T6	−1	14
AlSi8Cu3-B-T6-VF	VF	T6	−1	14
G12MnMo7-4+QT-POL	POL	QT	−1	16
G12MnMo7-4+QT-VF	VF	QT	−1	14
G21Mn5+N-POL	POL	N	−1	11
G21Mn5+N-VF	VF	N	−1	11

**Table 4:** Fatigue strength results for AlSi8Cu3 cast material.

Series’ Code	HT [−]	Surface Finish [−]	$\Delta \sigma_{\mathrm{LLF},50\%}$ [MPa]	$k_{1}$ [−]	$1:T_{S}$ [−]
AlSi8Cu3-A-HIP+T6-VF	HIP+T6	VF	135.1	8.3	1.11
AlSi8Cu3-A-HIP+T6-POL	HIP+T6	POL	122.6	8.9	1.27
AlSi8Cu3-A-T6-VF	T6	VF	123.9	5.8	1.08
AlSi8Cu3-A-T6-POL	T6	POL	94.7	4.8	1.22
AlSi8Cu3-B-T6-VF	T6	VF	62.8	5.4	1.29
AlSi8Cu3-B-T6-POL	T6	POL	54.1	5.1	1.14

**Table 5:** Comparison of fatigue results for cast steel series G21Mn5+Nand G12MnMo7-4+QT in polished a vibratory finished condition.

Series’ Code	HT [−]	Surface Finish [−]	$\Delta \sigma_{\mathrm{LLF},50\%}$ [MPa]	$k_{1}$ [−]	$1:T_{S}$ [−]
G12MnMo7-4+QT-VF	QT	VF	316.0	8.35	1.04
G12MnMo7-4+QT-POL	QT	POL	309.1	8.0	1.27
G21Mn5+N-VF	N	VF	219.4	16.0	1.12
G21Mn5+N-POL	N	POL	204.9	14.8	1.09

**Table 6:** Generalized R-curve parameters for cast steel alloy G12MnMo7-4+QT for $P_{Occ}=50\%$.

R[−]	$l_{1}$ [mm]	$l_{2}$ [mm]	$\nu_{1}$ [–]	$\nu_{2}$ [–]
−	0.02	1.0	0.4	0.6

**Table 7:** Long Crack growth threshold values $\Delta K_{th,lc}$ of series G12MnMo7-4+QT for $P_{Occ}=50\%$.

$R=-1\;[\mathrm{MPa}\sqrt{m}]$	$R=0\;[\mathrm{MPa}\sqrt{m}]$	$R=0.5\;[\mathrm{MPa}\sqrt{m}]$	$R=0.8\;[\mathrm{MPa}\sqrt{m}]$
13.6	7.6	3.4	2.71

**Table 8:** Summary of basic elastic–plastic parameters of the investigated cast alloys.

Series’ Code	$E^{\prime }\;$ [GPa]	$n^{\prime }\;\left[-\right]$	$K^{\prime }\;$ [MPa]	b[−]
AlSi8Cu3-A-HIP+T6	74.8	0.13	579	−0.079
AlSi8Cu3-B-HIP+T6	75.4	0.16	722	−0.099
G21Mn5+N	203.8	0.17	970	−0.096
G12MnMo7-4+QT	202.6	0.09	892	−0.069

**Table 9:** Non-linear combined hardening models for the investigated cast materials.

Series’ Code	k [MPa]	Q [MPa]	$b_{s}\;\left[-\right]$	$C_{1}\;\left[\mathrm{MPa}\right]$	$\gamma_{1}\;\left[-\right]$	$C_{2}\;\left[\mathrm{MPa}\right]$	$\gamma_{2}\;\left[-\right]$
AlSi8Cu3-A-HIP+T6	137.9	10.0	16.3	100,000	2342	43,949	157.9
AlSi8Cu3-B-HIP+T6	126.7	16	4.7	48,231	2040	68,161	351.2
G21Mn5+N	207.4	−14	13	108,100	1110	18,516	138
G12MnMo7-4+QT	351	−79	100	481,370	12,938	53,828	221

**Table 10:** GEV distribution parameters of the investigated fracture surfaces.

Series’ Code	μ[μm]	δ[μm]	ξ[−]	${\sqrt{\mathrm{area}}}_{\mathrm{Pocc}=50\%}\;\left[\mu m\right]$
AlSi8Cu3-A+T6	91.8	24.8	0.08	101.0
AlSi8Cu3-B+T6	377.0	76.5	0.1	405.6
G21Mn5+N	239.7	146.1	0.09	279.2
G12MnMo7-4+QT	114.0	40.2	−0.53	127.4

**Table 11:** Distribution parameters of shrinkage porosity over the entire specimen cross section analysed by CT.

Series’ Code	μ[μm]	δ[μm]	ξ[−]	${\sqrt{\mathrm{area}}}_{\mathrm{Pocc}=50\%}\;\left[\mu m\right]$
AlSi8Cu3-A+T6	108.7	20.5	0.28	116.6
AlSi8Cu3-B+T6	423.6	85.8	0.24	456.4

**Table 12:** Measured initial surface residual stress for polished and vibratory finished specimens.

Surface	AlSi8Cu3-A-HIP+T6 [MPa]	AlSi8Cu3-A-T6 [MPa]	G21Mn5+N [MPa]	G12MnMo7-4+QT [MPa]
POL	−79	−64	−221	−205
VF	−153	−119	−174	−314

**Table 13:** Stabilized effective load–stress ratio $R_{eff}$ in the specimen cross section determined by elastic–plastic FE simulations.

Position	Surface		$a_{0}/2$		$a_{0}$	
**Series**	**VF** [−]	**POL** [−]	**VF** [−]	**POL** [−]	**VF** [−]	**POL** [−]
AlSi8Cu3-A-HIP+T6	−3.1	−2.3	−2.3	−1.9	−1.8	−1.6
AlSi8Cu3-A-T6	−5.2	−6.5	−2.5	−1.1	−1.7	−1.0
AlSi8Cu3-B-T6	3.4	5.4	−1.5	−1.0	−0.9	−1.0
G12MnMo7-4+QT	−2.5	−2.2	−1.2	−1.0	−1.0	−1.0
G21Mn5+N	−1.9	−2.8	−1.3	−1.0	−1.1	−1.0

**Table 14:** Calculated fatigue strength of investigated series without effect of surface layer treatment ($R_{eff}=R$).

Surface	R[−]	$\sigma_{\mathrm{LLF},R-\mathrm{curve}}$ [MPa]	$\sigma_{\mathrm{LLF},\mathrm{EL}-\mathrm{Haddad}}$ [MPa]
AlSi8Cu3-LS-T6	−1	82.3	87.8
AlSi8Cu3-ST-T6	−1	65.2	70.6
G12MnMo7-4+QT	−1	280.7	331.5
G21Mn5+N	−1	206.5	199.1

**Table 15:** Calculated long life fatigue strength values $\sigma_{LLF,50\%}$ focussing on the effect of $R_{eff}$ compared to the experimental results using different surface treatments.

Series	Experiment		R-Curve				EL-Haddad			
	$\sigma_{\mathrm{LLF},\mathrm{POL}}$ [MPa]	$\sigma_{\mathrm{LLF},\mathrm{VF}}$ [MPa]	$\sigma_{\mathrm{LLF},\mathrm{POL}}$ [MPa]	$\sigma_{\mathrm{LLF},\mathrm{VF}}$ [MPa]	Δ POL [%]	Δ VF [%]	$\sigma_{\mathrm{LLF},\mathrm{POL}}$ [MPa]	$\sigma_{\mathrm{LLF},\mathrm{VF}}$ [MPa]	Δ POL [%]	Δ VF [%]
AlSi8Cu3-A-T6	94.7	123.9	82.1	114.6	13.3	7.5	84.9	115.5	10.3	6.8
AlSi8Cu3-B-T6	54.1	62.8	58.4	69.7	−7.9	−11.0	66.9	76.8	−23.7	−22.3
G12MnMo7-4+QT	309.1	316.0	280.7	297.3	9.2	5.9	331.5	346.9	−7.2	−9.8
G21Mn5+N	204.9	219.4	206.5	231.0	−0.8	−5.3	199.1	214.0	2.8	2.5

**Table 16:** Calculated fatigue strength of Murakami’s approach compared to experimental results.

Series	Experiment		Murakami			
	$\sigma_{\mathrm{LLF},\mathrm{POL}}$ **[MPa]**	$\sigma_{\mathrm{LLF},\mathrm{VF}}$ **[MPa]**	$\sigma_{\mathrm{LLF},\mathrm{POL}}$ **[MPa]**	$\sigma_{\mathrm{LLF},\mathrm{VF}}$ **[MPa]**	Δ **POL [%]**	Δ **VF [%]**
AlSi8Cu3-A-T6	94.7	123.9	109.1	141.2	−15.5	−14.0
AlSi8Cu3-B-T6	54.1	62.8	86.2	93.1	−59.3	−48.2
G12MnMo7-4+QT	309.1	316.0	229.6	243.9	25.7	22.8
G21Mn5+N	204.9	219.4	175.4	195.7	14.4	10.8

## Data Availability

Not applicable.

## Abbreviations

The following abbreviations are used in this manuscript:

$\sqrt{\mathrm{area}}$	Defect size perpendicular to the load direction
$\gamma_{c}$	Crack opening function
$\gamma_{i}$	Kinematic material parameter
δ	Scale parameter of the GEV distribution
Δ	Deviation in % between two values
$\Delta \sigma_{0}$	Fatigue range of defect free material
$\Delta K_{th,eff}$	Effective crack threshold range
$\Delta K_{th,lc}$	Long crack threshold range
$\Delta K_{th,lc,R0}$	Long crack threshold value at tumescent loading
$\epsilon_{a,el}$	Elastic strain amplitude
$\epsilon_{a,pl}$	Plastic strain amplitude
$\epsilon_{a,t}$	Total strain amplitude
$\epsilon_{f}^{\prime }$	Fatigue ductility coefficient
$\epsilon_{p}$	Plastic strain
$\epsilon_{p,0}$	Initial plastic strain
$\lambda_{2}$	Secondary dendrite arm spacing
μ	Location parameter of the GEV distribution
$\nu_{i}$	Weighting factor for crack closure
ξ	Shape parameter of the GEV distribution
σ	Stress tensor
$\sigma^{\prime }$	Deviatoric part of the stress tensor
$\sigma_{a}$	Stress amplitude
$\sigma_{f}^{\prime }$	Fatigue strength coefficient
$\sigma_{LLF}$	Long life fatigue strength at ten million load cycles
$\sigma_{res,eff}$	Effective, cyclic stable residual stresses
$\sigma_{zz,eff}^{a_{0}}$	Cyclic stable residual stresses at distance $a_{0}$ from surface
$\sigma_{zz,eff}^{a_{0}/2}$	Cyclic stable residual stresses at distance $a_{0}/2$ from surface
$\sigma_{zz,eff}^{\mathrm{center}}$	Cyclic stable residual stresses at specimen center
$\sigma_{zz}^{res}$	Residual stresses in z-direction
$\sigma_{zz,eff}^{\mathrm{surf}}$	Cyclic stable residual stresses at surface
χ	Kinematic back stress tensor
$\chi^{\prime }$	Deviatoric part of kinematic back stress tensor
ψ	Direction of flow
*a*	Crack length
$a_{0}$	EL Haddad constant
$a_{0,eff}$	Intrinsic crack length
$A_{0}$	Newman coefficient
*b*	Fatigue strength exponent
$b_{s}$	Stabilization rate of yield surface
*c*	Fatigue ductility exponent
$C_{i}$	Kinematic material parameter
$C_{th}$	Material constant of Newman curve
*E*	Quasistatic Young’s Modulus
*f*	Yield function
HV	Vickers hardness
*I*	Isotropic part of combined hardening model
$J(\sigma -\chi^{\prime })$	von Mises criterion
*k*	Initial size of the yield function
$k_{1}$	Inverse slope of the S/N-curve in finite life region
$k_{2}$	Inverse slope of the S/N-curve in long life region
$K^{\prime }$	Cyclic strength coefficient
$l_{i}$	Crack elongation where closure effect $\nu_{i}$ is completely developed
LYS	Lower yield strength
$n^{\prime }$	Cyclic strain hardening exponent
*p*	Accumulated plastic strain
$P_{occ}$	Probability of occurrence
$P_{s}$	Probability of survival
*Q*	Difference between initial and stabilized size of the yield surface
$R_{\epsilon }$	Strain ratio
*R*	Load stress ratio
$R_{a}$	Arithmetic mean high of the roughness profile
$R_{z}$	Mean high of the roughness profile elements
$R_{eff}$	Effective cyclic stable load ratio
TS	Tensile strength
$T_{s}$	Fatigue scatter index of the S/N-curve
UTS	Ultimate tensile strength
UYS	Upper yield strength
*Y*	Geometry factor
YS	Yield strength
CT	Computed tomography
DCPD	Direct current potential drop
DOM	Digital optical microscope
EDM	Electrical discharge machining
FKM	Forschungskuratorium Maschinenbau
GEV	Generalized extreme value distribution
HCF	High cycle fatigue
HIP	Hot isostatic pressed
HSV	Highly stressed volume
KS	Kolmogorov-Smirnov
KTD	Kitagawa–Takahashi diagram
LCF	Low cycle fatigue
POL	Polished surface state
POT	Peak over threshold sampling
SEM	Scanning electron microscope
TCD	Theory of critical distances
VF	Vibratory finished surface state
XRD	X-ray diffraction analysis
